# Multimodal Hierarchical Dirichlet Process-Based Active Perception by a Robot

**DOI:** 10.3389/fnbot.2018.00022

**Published:** 2018-05-22

**Authors:** Tadahiro Taniguchi, Ryo Yoshino, Toshiaki Takano

**Affiliations:** ^1^Emergent Systems Laboratory, College of Information Science and Engineering, Ritsumeikan University, Ksatsu Japan; ^2^Adaptive Systems Laboratory, Department of Computer Science, Shizuoka Institute of Science and Technology, Fukuroi, Japan

**Keywords:** active perception, cognitive robotics, topic model, multimodal machine learning, submodular maximization

## Abstract

In this paper, we propose an active perception method for recognizing object categories based on the multimodal hierarchical Dirichlet process (MHDP). The MHDP enables a robot to form object categories using multimodal information, e.g., visual, auditory, and haptic information, which can be observed by performing actions on an object. However, performing many actions on a target object requires a long time. In a real-time scenario, i.e., when the time is limited, the robot has to determine the set of actions that is most effective for recognizing a target object. We propose an active perception for MHDP method that uses the information gain (IG) maximization criterion and lazy greedy algorithm. We show that the IG maximization criterion is optimal in the sense that the criterion is equivalent to a minimization of the expected Kullback–Leibler divergence between a final recognition state and the recognition state after the next set of actions. However, a straightforward calculation of IG is practically impossible. Therefore, we derive a Monte Carlo approximation method for IG by making use of a property of the MHDP. We also show that the IG has submodular and non-decreasing properties as a set function because of the structure of the graphical model of the MHDP. Therefore, the IG maximization problem is reduced to a submodular maximization problem. This means that greedy and lazy greedy algorithms are effective and have a theoretical justification for their performance. We conducted an experiment using an upper-torso humanoid robot and a second one using synthetic data. The experimental results show that the method enables the robot to select a set of actions that allow it to recognize target objects quickly and accurately. The numerical experiment using the synthetic data shows that the proposed method can work appropriately even when the number of actions is large and a set of target objects involves objects categorized into multiple classes. The results support our theoretical outcomes.

## 1. Introduction

**Figure 1:**
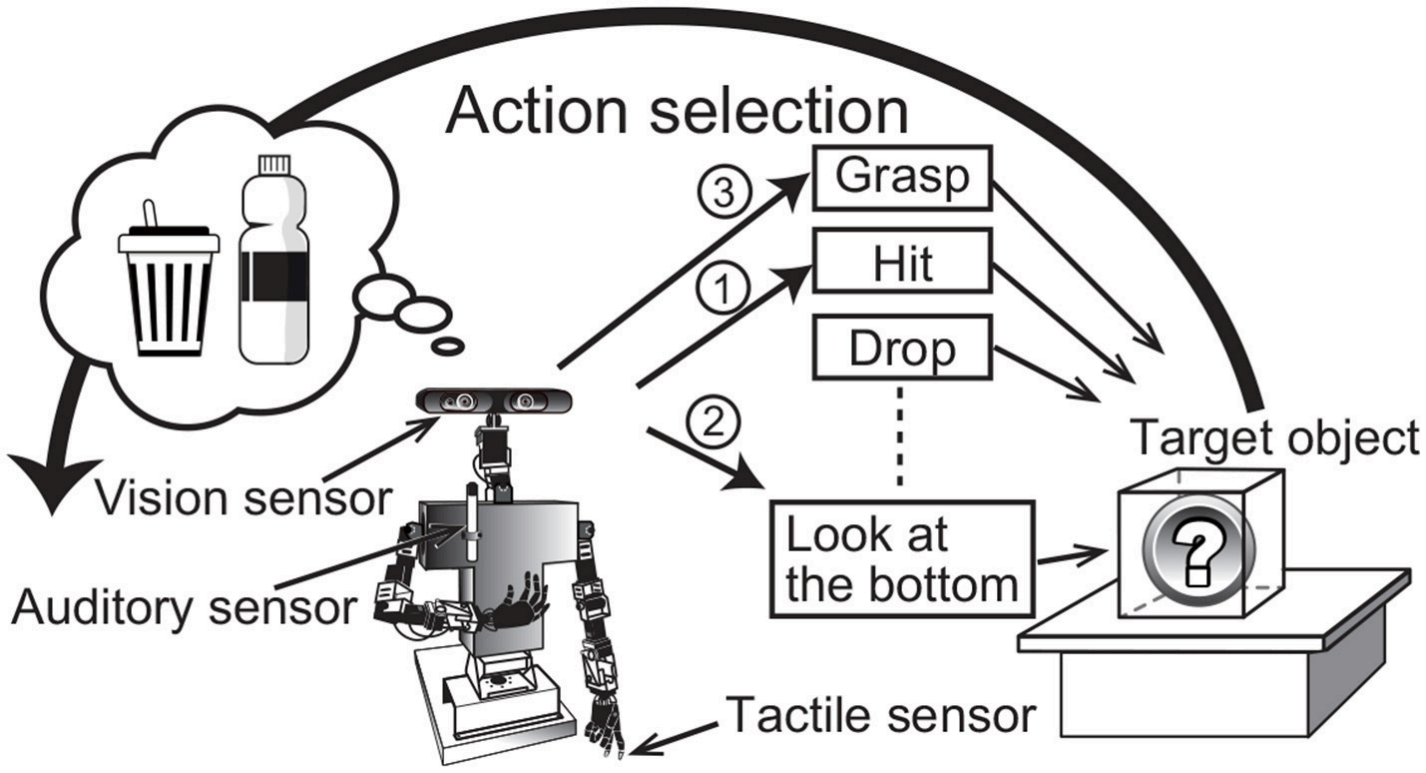
Overview of active perception for multimodal object category recognition. The numbers attached to the arrows show a sample of the order of action selection by the robot.

Active perception is a fundamental component of our cognitive skills. Human infants autonomously and spontaneously perform actions on an object to determine its nature. The sensory information that we can obtain usually depends on the actions performed on the target object. For example, when people find a gift box placed in front of them, they cannot perceive its weight without holding the box, and they cannot determine its sound without hitting or shaking it. In other words, we can obtain sensory information about an object by selecting and executing actions to manipulate it. Adequate action selection is important for recognizing objects quickly and accurately. This example about a human also holds for a robot. An autonomous robot that moves and helps people in a living environment should also select adequate actions to recognize target objects. For example, when a person asks an autonomous robot to bring an empty plastic bottle, the robot has to examine many objects by applying several actions (Figure 1). This type of information is important, because our object categories are formed on the basis of multimodal information, i.e., not only visual information is used, but also auditory, haptic, and other information. Therefore, a computational model of the active perception should be consistently based on a computational model for multimodal object categorization and recognition.

In spite of the wide range of studies about active perception (e.g., Borotschnig et al., 2000; Dutta Roy et al., 2004; Eidenberger and Scharinger, 2010; Krainin et al., 2011; Ferreira et al., 2013) and multimodal categorization for robots (e.g., Nakamura et al., 2007, 2011a; Sinapov and Stoytchev, 2011; Celikkanat et al., 2014; Sinapov et al., 2014), active perception methods for a robot, i.e., action selection methods for perception for unsupervised multimodal categorization, have not been sufficiently explored (see section 2).

This paper considers the active perception problem for unsupervised multimodal object categorization under the condition that a robot has already obtained several action primitives that are used to examine target objects. In the context of this study, we need to study active perception on an unsupervised multimodal categorization method having generality as much as possible because it is believed that unsupervised multimodal categorization is important for future language learning by robots, and the findings obtained in this study should be able to be applied to other unsupervised multimodal categorization models. It was suggested that a child forms a category based on his/her sensorimotor experience before learning a word for the category in a Bayesian manner, and learning the word is a matter of attaching a new label to this preexisting category (Kemp et al., 2010). The multimodal hierarchical Dirichlet process (MHDP) is a mathematically very general and sophisticated nonparametric Bayesian multimodal categorization method. Therefore, we adopt MHDP proposed by Nakamura et al. (2011b) as a representative computational model for unsupervised multimodal object categorization.

We develop an active perception method based on the MHDP in this paper. The MHDP is a sophisticated, fully Bayesian, probabilistic model for multimodal object categorization (Nakamura et al., 2011b) that is developed by enabling hierarchical Dirichlet process (HDP) (Teh et al., 2006) to have multimodal emission distributions corresponding to multiple sensor information^1^. Nakamura et al. (2011b) showed that the MHDP enables a robot to form object categories using multimodal information, i.e., visual, auditory, and haptic information, in an unsupervised manner. The MHDP can estimate the number of object categories as well because of the nature of Bayesian nonparametrics.

This paper describes a new MHDP-based active perception method for multimodal object recognition based on object categories formed by a robot itself. We found that an active perception method that has a good theoretical nature, i.e., the performance of the greedy algorithm is theoretically guaranteed (see section 4), can be derived for MHDP. Our formulation is based on a hierarchical Bayesian model. If a cognitive system of a robot is modeled by using hierarchical Bayesian model, a recognition state are usually represented by posterior distribution over latent variables, e.g., object categories. The purpose of an active perception is to infer appropriate posterior distribution with a small number of actions. In our approach, we propose an action selection method that can reduce the distance between inferred posterior distributions and true posterior distributions.

In this study, we define the active perception problem in the context of unsupervised multimodal object categorization as following. Which set of actions should a robot take to recognize a target object as accurately as possible under the constraint that the number of actions is restricted^2^? Our MHDP-based active perception method uses an IG maximization criterion, Monte Carlo approximation, and the lazy greedy algorithm. In this paper, we show that the MHDP provides the following three advantages for deriving an efficient active perception method.

1. The *IG maximization criterion* is *optimal* in the sense that a selected set of actions minimizes the expected Kullback–Leibler (KL) divergence between the final posterior distribution estimated using the information regarding all modalities and the posterior distribution of the category estimated using the selected set of actions (see section 4.1).
2. The IG has a *submodular* and non-decreasing property as a set function. Therefore, for performance, the greedy and lazy greedy algorithms are guaranteed to be near-optimal strategies (see section 4.2).
3. A *Monte Carlo approximation* method for the IG can be derived by exploiting MHDP's properties (see section 4.3).

Although the above properties follow from the theoretical characteristics of the MHDP, this has never been pointed out in previous studies.

The main contributions of this paper are that we

- develop an MHDP-based active perception method, and
- show its effectiveness through experiments using an upper-torso humanoid robot and synthetic data.

The proposed active perception method can be used for general purposes, i.e., not only for robots but also for other target domains to which the MHDP can be applied. In addition, The proposed method can be easily extended for other multimodal categorization methods with similar graphical models, e.g., multimodal latent Dirichlet allocation (MLDA) (Nakamura et al., 2009). However, in this paper, we focus on the MHDP and the robot active perception scenario, and explain our method on the basis of this task.

The remainder of this paper is organized as follows. Section 2 describes the background and work related to our study. Section 3 briefly introduces the MHDP, proposed by Nakamura et al. (2011b), which enables a robot to obtain object categories by fusing multimodal sensor information in an unsupervised manner. Section 4 describes our proposed action selection method. Section 5 discusses the effectiveness of the action selection method through experiments using an upper-torso humanoid robot. Section 6 describes a supplemental experiment using synthetic data. Section 7 concludes this paper.

## 2. Background and related work

### 2.1. Multimodal categorization

The human capability for object categorization is a fundamental topic in cognitive science (Barsalou, 1999). In the field of robotics, adaptive formation of object categories that considers a robot's embodiment, i.e., its sensory-motor system, is gathering attention as a way to solve the symbol grounding problem (Harnad, 1990; Taniguchi et al., 2016).

Recently, various computational models and machine learning methods for multimodal object categorization have been proposed in artificial intelligence, cognitive robotics, and related research fields (Roy and Pentland, 2002; Natale et al., 2004; Nakamura et al., 2007, 2009, 2011a,b, 2014; Iwahashi et al., 2010; Sinapov and Stoytchev, 2011; Araki et al., 2012; Griffith et al., 2012; Ando et al., 2013; Celikkanat et al., 2014; Sinapov et al., 2014). For example, Sinapov and Stoytchev (2011) proposed a graph-based multimodal categorization method that allows a robot to recognize a new object by its similarity to a set of familiar objects. They also built a robotic system that categorizes 100 objects from multimodal information in a supervised manner (Sinapov et al., 2014). Celikkanat et al. (2014) modeled the context in terms of a set of concepts that allow many-to-many relationships between objects and contexts using LDA.

Our focus of this paper is not a supervised learning-based, but an unsupervised learning-based multimodal categorization method and an active perception method for categories formed by the method. Of these, a series of statistical unsupervised multimodal categorization methods for autonomous robots have been proposed by extending LDA, i.e., a topic model (Nakamura et al., 2007, 2009, 2011a,b, 2014; Araki et al., 2012; Ando et al., 2013). All these methods are Bayesian generative models, and the MHDP is a representative method of this series (Nakamura et al., 2011b). The MHDP is an extension of the HDP, which was proposed by Teh et al. (2006), and the HDP is a nonparametric Bayesian extension of LDA (Blei et al., 2003). Concretely, the generative model of the MHDP has multiple types of emissions that correspond to various sensor data obtained through various modality inputs. In the HDP, observation data are usually represented as a bag-of-words (BoW). In contrast, the observation data in the MHDP use bag-of-features (BoF) representations for multimodal information. BoF is a histogram-based feature representation that is generated by quantizing observed feature vectors. Latent variables that are regarded as indicators of *topics* in the HDP correspond to *object categories* in the MHDP. Nakamura et al. (2011b) showed that the MHDP enables a robot to categorize a large number of objects in a home environment into categories that are similar to human categorization results.

To obtain multimodal information, a robot has to perform actions and interact with a target object in various ways, e.g., grasping, shaking, or rotating the object. If the number of actions and types of sensor information increase, multimodal categorization and recognition can require a longer time. When the recognition time is limited and/or if quick recognition is required, it becomes important for a robot to select a small number of actions that are effective for accurate recognition. Action selection for recognition is often called active perception. However, an active perception method for the MHDP has not been proposed. This paper aims to provide an active perception method for the MHDP.

### 2.2. Active perception

Generally, active perception is one of the most important cognitive capabilities of humans. From an engineering viewpoint, active perception has many specific tasks, e.g., localization, mapping, navigation, object recognition, object segmentation, and self–other differentiation.

In machine learning, *active learning* is defined as a task in which a method interactively queries an information source to obtain the desired outputs at new data points to learn efficiently Settles (2012). Active learning algorithms select an unobserved input datum and ask a user (labeler) to provide a training signal (label) in order to reduce uncertainty as quickly as possible (Cohn et al., 1996; Muslea et al., 2006; Settles, 2012). These algorithms usually assume a supervised learning problem. This problem is related to the problem in this paper, but is fundamentally different.

Historically, active vision, i.e., active visual perception, has been studied as an important engineering problem in computer vision. Dutta Roy et al. (2004) presented a comprehensive survey of active three-dimensional object recognition. For example, Borotschnig et al. (2000) proposed an active vision method in a parametric eigenspace to improve the visual classification results. Denzler and Brown (2002) proposed an information theoretic action selection method to gather information that conveys the true state of a system through an active camera. They used the mutual information (MI) as a criterion for action selection. Krainin et al. (2011) developed an active perception method in which a mobile robot manipulates an object to build a three-dimensional surface model of it. Their method uses the IG criterion to determine when and how the robot should grasp the object.

Modeling and/or recognizing a single object as well as modeling a scene and/or segmenting objects are also important tasks in the context of robotics. Eidenberger and Scharinger (2010) proposed an active perception planning method for scene modeling in a realistic environment. van Hoof et al. (2012) proposed an active scene exploration method that enables an autonomous robot to efficiently segment a scene into its constituent objects by interacting with the objects in an unstructured environment. They used IG as a criterion for action selection. InfoMax control for acoustic exploration was proposed by Rebguns et al. (2011).

Localization, mapping, and navigation are also targets of active perception. Velez et al. (2012) presented an online planning algorithm that enables a mobile robot to generate plans that maximize the expected performance of object detection. Burgard et al. (1997) proposed an active perception method for localization. Action selection is performed by maximizing the weighted sum of the expected entropy and expected costs. To reduce the computational cost, they only consider a subset of the next locations. Roy and Thrun (1999) proposed a coastal navigation method for a robot to generate trajectories for its goal by minimizing the positional uncertainty at the goal. Stachniss et al. (2005) proposed an information-gain-based exploration method for mapping and localization. Correa and Soto (2009) proposed an active perception method for a mobile robot with a visual sensor mounted on a pan-tilt mechanism to reduce localization uncertainty. They used the IG criterion, which was estimated using a particle filter.

In addition, various studies on active perception by a robot have been conducted (Natale et al., 2004; Ji and Carin, 2006; Schneider et al., 2009; Tuci et al., 2010; Saegusa et al., 2011; Fishel and Loeb, 2012; Pape et al., 2012; Sushkov and Sammut, 2012; Gouko et al., 2013; Hogman et al., 2013; Ivaldi et al., 2014; Zhang et al., 2017). In spite of a large number of contributions about active perception, few theories of active perception for multimodal object category recognition have been proposed. In particular, an MHDP-based active perception method has not yet been proposed, although the MHDP-based categorization method and its series have obtained many successful results and extensions.

### 2.3. Active perception for multimodal categorization

Sinapov et al. (2014) investigated multimodal categorization and active perception by making a robot perform 10 different behaviors; obtain visual, auditory, and haptic information; explore 100 different objects, and classify them into 20 object categories. In addition, they proposed an active behavior selection method based on confusion matrices. They reported that the method was able to reduce the exploration time by half by dynamically selecting the next exploratory behavior. However, their multimodal categorization is performed in a supervised manner, and the theory of active perception is still heuristic. The method does not have theoretical guarantees of performance.

IG-based active perception is popular, as shown above, but the theoretical justification for using IG in each task is often missing in many robotics papers. Moreover, in many cases in robotics studies, IG cannot be evaluated directly, reliably, or accurately. When one takes an IG criterion-based approach, how to estimate the IG is an important problem. In this study, we focus on MHDP-based active perception and develop an efficient near-optimal method based on firm theoretical justification.

## 3. Multimodal hierarchical dirichlet process for statistical multimodal categorization

**Figure 2:**
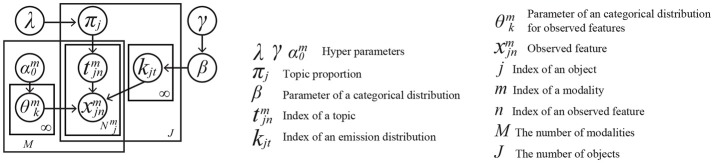
Graphical representation of an MHDP with *M* modalities corresponding to actions for perception.

We assume that a robot forms object categories using the MHDP from multimodal sensory data. In this section, we briefly introduce the MHDP on which our proposed active perception method is based (Nakamura et al., 2011b). The MHDP assumes that an observation node in its graphical model corresponds to an action and its corresponding modality. Nakamura et al. (2011b) employed three observation nodes in their graphical model, i.e., haptic, visual, and auditory information nodes. Three actions, i.e., grasping, looking around, and shaking, correspond to these modalities, respectively. However, the MHDP can be easily extended to a model with additional types of sensory inputs. It is without doubt that autonomous robots will also gain more types of action for perception. For modeling more general cases, an MHDP with *M* actions is described in this paper. A graphical model of the MHDP is illustrated in Figure 2. In this section, we describe the MHDP briefly. For more details, please refer to Nakamura et al. (2011b).

The index *m* ∈ **M** (#(**M**) = *M*) in Figure 2 represents the type of information that corresponds to an action for perception, e.g., hitting an object to obtain its sound, grasping an object to test its shape and hardness, or looking at all of an object by rotating it. We assume that a robot has action primitives and it can execute one of the actions by selecting the index of the action primitives. The observation $x_{jn}^{m}\in X^{m}$ is the *m*-th modality's *n*-th feature for the *j*-th target object. *X*^*m*^ represents a set of observation of *m*-th modality. The observation $x_{jn}^{m}$ is assumed to be drawn from a categorical distribution whose parameter is $\theta_{k}^{m}$, where *k* is an index of a latent topic. Each index *k* is drawn from a categorical distribution whose parameter is β that is drawn from a Dirichlet distribution parametrized by γ. Parameter $\theta_{k}^{m}$ is assumed to be drawn from the Dirichlet prior distribution whose parameter is $\alpha_{0}^{m}$. The MHDP assumes that a robot obtains each modality's sensory information as a BoF representation. Each latent variable $t_{jn}^{m}$ is drawn from a topic proportion, i.e., a parameter of a multinomial distribution, of the *j*-th object π_*j*_ whose prior is a Dirichlet distribution parametrized by λ.

Similarly to the generative process of the original HDP (Teh et al., 2006), the generative process of the MHDP can be described as a Chinese restaurant franchise, which is the name of a special type of probabilistic process in Bayesian nonparametrics (Teh et al., 2005). The learning and recognition algorithms are both derived using Gibbs sampling. In its learning process, the MHDP estimates a latent variable $t_{jn}^{m}$ for each feature of the *j*-th object and a topic index *k*_*jt*_ for each latent variable *t*. The combination of latent variable and topic index corresponds to a topic in LDA (Blei et al., 2003). Using the estimated latent variables, the categorical distribution parameter $\theta_{k}^{m}$ and topic proportion of the *j*-th object π_*j*_ are drawn from the posterior distribution.

The selection procedure for latent variable $t_{jn}^{m}$ is as follows. The prior probability that $x_{jn}^{m}$ selects *t* is

$$\begin{array}{l} P(t_{jn}^{m}=t|\lambda )=\{\begin{array}{ll} \frac{\sum_{m}w^{m}N_{jt}^{m}}{\lambda +\sum_{m}w^{m}N_{j}^{m}-1}, & (t=1,\cdots ,T_{j}),\\ \frac{\lambda }{\lambda +\sum_{m}w^{m}N_{j}^{m}-1}, & (t=T_{j}+1), \end{array} \end{array}$$

where *w*^*m*^ is a weight for the *m*-th modality, To balance the influence of different modalities, *w*^*m*^ are set as hyperparameters. The weight *w*^*m*^ increases the influence of the modality *m* on multimodal category formation. $N_{jt}^{m}$ is the number of *m*-th modality observations that are allocated to *t* in the *j*-th object, and λ is a hyperparameter. In the Chinese restaurant process, if the number of observed features $N_{jt}=\underset{m}{\sum }w^{m}N_{jt}^{m}$ that are allocated to *t* increases, the probability at which a new observation is allocated to the latent variable *t* increases. Using the prior distribution, the posterior probability that observation $x_{jn}^{m}$ is allocated to the latent variable *t* becomes

$$\begin{array}{l} P(t_{jn}^{m}=t|X^{m},\lambda )=\frac{P(x_{jn}^{m}|X_{k\;=\;k_{jt}}^{m})P(t_{jn}^{m}=t|\lambda )}{P(x_{jn}^{m}|X^{m}\setminus \{x_{jn}^{m}\},\lambda )}\\ \;\propto \{\begin{array}{ll} P(x_{jn}^{m}|X_{k\;=\;k_{jt}}^{m})\frac{\sum_{m}w^{m}N_{jt}^{m}}{\lambda +\sum_{m}w^{m}N_{j}^{m}-1}, & (t=1,\cdots ,T_{j}),\\ P(x_{jn}^{m}|X_{k\;=\;k_{jt}}^{m})\frac{\lambda }{\lambda +\sum_{m}w^{m}N_{j}^{m}-1}, & (t=T_{j}+1), \end{array} \end{array}$$

where $N_{j}^{m}$ is the number of the *m*-th modality's observations about the *j*-th object. The set of observations that correspond to the *m*-th modality and have the *k*-th topic in any object are represented by $X_{k}^{m}$.

In the Gibbs sampling procedure, a latent variable for each observation is drawn from the posterior probability distribution. If *t* = *T*_*j*_ + 1, a new observation is allocated to a new latent variable. The dish selection procedure is as follows. The prior probability that the *k*-th topic is allocated on the *t*-th latent variable becomes

$$\begin{array}{l} P(k_{jt}=k|\gamma )=\{\begin{array}{ll} \frac{M_{k}}{\gamma \;+\;M-1}, & (k=1,\cdots ,K),\\ \frac{\gamma }{\gamma \;+\;M-1}, & (k=K+1), \end{array} \end{array}$$

where *K* is the number of topic types, and *M*_*k*_ is the number of latent variables on which the *k*-th topic is placed. Therefore, the posterior probability that the *k*-th topic is allocated on the *t*-th latent variable becomes

$$\begin{array}{l} P(k_{jt}=k|X,\gamma )=P(X_{jt}|X_{k})P(k_{jt}=k|\gamma )\\ \;=\{\begin{array}{ll} P(X_{jt}|X_{k})\frac{M_{k}}{\gamma \;+\;M-1}, & (k=1,\cdots ,K),\\ P(X_{jt}|X_{k})\frac{\gamma }{\gamma \;+\;M-1}, & \left(k=K+1\right) \end{array} \end{array}$$

where $X=\cup_{m}X^{m}$, $X_{k}=\cup_{m}X_{k}^{m}$, and *X*_*jt*_ is the set of the *j*-th object's observations allocated to the *t*-th latent variable. A topic index for the latent variable *t* for the *j*-th object is drawn using the posterior probability, where γ is a hyperparameter. If *k* = *K* + 1, a new topic is placed on the latent variable.

By sampling $t_{jn}^{m}$ and *k*_*jt*_, the Gibbs sampler performs probabilistic object clustering:

(1) $$\begin{array}{l} t_{jn}^{m}\sim P(t_{jn}^{m}|X^{-mjn},\lambda ), \end{array}$$

(2) $$\begin{array}{l} k_{jt}\sim P(k_{jt}|X^{-jt},\gamma ), \end{array}$$

where $X^{-mjn}=X\backslash \left\{x_{jn}^{m}\right\}$, and $X^{-jt}=X\backslash X_{jt}$. By sampling $t_{jn}^{m}$ for each observation in every object using (1) and sampling *k*_*jt*_ for each latent variable *t* in every object using (2), all of the latent variables in the MHDP can be inferred.

If $t_{jn}^{m}$ and *k*_*jt*_ are given, the probability that the *j*-th object is included in the *k*-th category becomes

(3) $$\begin{array}{l} P(k|X_{j})=\frac{\Sigma_{t\;=\;1}^{T_{j}}\delta_{k}(k_{jt})\sum_{m}w^{m}N_{jt}^{m}}{\sum_{m}w^{m}N_{j}^{m}}, \end{array}$$

where $X_{j}=\cup_{m}X_{j}^{m}$, *w*^*m*^ is the weight for the *m*-th modality and δ_*a*_(*x*) is a delta function.

When a robot attempts to recognize a new object after the learning phase, the probability that feature $x_{jn}^{m}$ is generated from the *k*-th topic becomes

$$\begin{array}{l} P(x_{jn}^{m}|X_{k}^{m})=\frac{w^{m}N_{kx_{jn}^{m}}^{m}+\alpha_{0}^{m}}{w^{m}N_{k}^{m}+d^{m}\alpha_{0}^{m}}, \end{array}$$

where *d*^*m*^ denotes the dimension of the *m*-th modality input, and $N_{kx_{jn}^{m}}^{m}$ represents the number of features $x_{jn}^{m}$ that is corresponding to the index *k*. Topic *k*_*t*_ allocated to *t* for a new object is sampled from

$$\begin{array}{l} k_{t}\sim P(k_{jt}=k|X,\gamma )\propto P(X_{jt}|X_{k})\frac{\gamma }{\gamma +M-1}. \end{array}$$

These sampling procedures play an important role in the Monte Carlo approximation of our proposed method (see section 4.2.).

For a more detailed explanation of the MHDP, please refer to Nakamura et al. (2011b). Basically, a robot can autonomously learn object categories and recognize new objects using the multimodal categorization procedure described above. The performance and effectiveness of the method was evaluated in the paper.

## 4. Active perception method

### 4.1. Basic formulation

A robot should have already conducted several actions and obtained information from several modalities when it attempts to select next action set for recognizing a target object. For example, visual information can usually be obtained by looking at the front face of the *j*-th object from a distance before interacting with the object physically. We assume that a robot has already obtained information corresponding to a subset of modalities *m*_*o*_*j*__ ⊂ **M**, where the subscript **o** means“originally” obtained modality information. When a robot faces a new object and has not obtained any information, *m*_*o*_*j*__ = ∅.

The purpose of object recognition in multimodal categorization is different from conventional supervised learning-based pattern recognition problems. In supervised learning, the recognition result is evaluated by checking whether the output is the same as the truth label. However, in unsupervised learning, there are basically no truth labels. Therefore, the performance of active perception should be measured in a different manner.

The action set the robot selects is described as $A=\{a_{1},a_{2},\ldots ,a_{N_{A}}\}\in 2^{M\setminus m_{o}{\;}_{j}}$, where $2^{M\setminus m_{o}{\;}_{j}}$ is a family of subsets of **M** \ *m*_*o*_*j*__, i.e., **A** ⊂ **M** \ *m*_*o*_*j*__, *a*_*i*_ ∈ **M** \ *m*_*o*_*j*__ and *N*_*A*_ represents the number of available actions. We consider an effective action set for active perception to be one that largely reduces the distance between the final recognition state after the information from all modalities **M** is obtained and the recognition state after the robot executes the selected action set **A**. The recognition state is represented by the posterior distribution $P(z_{j}|X_{j}^{m_{o}{\;}_{j}\cup^{\text{​}}A})$. Here, $z_{j}=\{{\left\{k_{jt}\right\}}_{1\leq t\leq T_{j}},\;{\left\{t_{jn}^{m}\right\}}_{m\in M,1\leq n\leq N_{j}^{m}}\}$ is a latent variable representing the *j*-th object's topic information, where $X_{j}^{A}=\cup_{m\in A}X_{j}^{m},X_{j}^{m}=\{x_{j1}^{m},\ldots ,x_{jn}^{m},\ldots ,x_{jN_{j}^{m}}^{m}\}$. Probability $P(z_{j}|X_{j}^{m_{o}{\;}_{j}\cup^{\text{​}}A})$ represents the posterior distribution related to the object category after taking actions *m*_*o*_*j*__ and **A**.

The final recognition state, i.e., posterior distribution over latent variables after obtaining the information from all modalities **M**, becomes $P\left({\text{z}}_{j}|X_{j}^{\text{M}}\right)$. The purpose of active perception is to select a set of actions that can estimate the posterior distribution most accurately. When *L* actions can be executed, if we employ KL divergence as the metric of the difference between the two probability distributions,

(4) $$\begin{array}{l} \underset{A\in F_{L}^{m_{o}{\;}_{j}}}{\text{minimize}}\text{ KL }(P(z_{j}|X_{j}^{M}),P(z_{j}|X_{j}^{m_{o}{\;}_{j}\cup A})) \end{array}$$

is a reasonable evaluation criterion for realizing effective active perception, where $F_{L}^{m_{o}{\;}_{j}}=\{A|A\subset M\setminus m_{o}{\;}_{j},N_{A}\leq L\}$ is a feasible set of actions.

However, neither the true $X_{j}^{M}\text{ nor }X_{j}^{m_{o}{\;}_{j}\cup A}$ can be observed before taking **A** on the *j*-th target object, and hence cannot be used at the moment of action selection. Therefore, a rational alternative for the evaluation criterion is the expected value of the KL divergence at the moment of action selection:

(5) $$\begin{array}{l} \underset{A\in F_{L}^{m_{o}{\;}_{j}}}{\text{minimize}}E_{X_{j}^{M\backslash m_{o}{\;}_{j}}|X_{j}^{m_{o}{\;}_{j}}}[\text{KL }(P(z_{j}|X_{j}^{M}),P(z_{j}|X_{j}^{m_{o}{\;}_{j}\cup A}))]. \end{array}$$

Here, we propose to use the IG maximization criterion to select the next action set for active perception:

(6) $$\begin{array}{l} A_{j}^{*}=\underset{A\in F_{L}^{m_{o}{\;}_{j}}}{\text{argmax}}\text{ IG }(z_{j};X_{j}^{A}|X_{j}^{m_{o}{\;}_{j}}) \end{array}$$

(7) $$\begin{array}{l} \;=\underset{A\in F_{L}^{m_{o}{\;}_{j}}}{\text{argmin}}E_{X_{j}^{A}|X_{j}^{m_{o}{\;}_{j}}}[\text{KL }(P(z_{j}|X_{j}^{m_{o}{\;}_{j}\cup A}),P(z_{j}|X_{j}^{m_{o}{\;}_{j}}))], \end{array}$$

where IG(*X*; *Y*|*Z*) is the IG of *Y* for *X*, which is calculated on the basis of the probability distribution commonly conditioned by *Z* as follows:

$$\begin{array}{l} \text{IG }(X;Y|Z)=\text{KL }(P(X,Y|Z),P(X|Z)P(Y|Z)). \end{array}$$

By definition, the expected KL divergence is the same as IG(*X*; *Y*). The definition of IG and its relation to KL divergence are as follows.

$$\begin{array}{l} \text{IG }(X;Y)=H(X)-H(X|Y)\\ \;=\text{KL }(P(X,Y),P(X)P(Y))\\ \;=E_{Y}[\text{KL }(P(X|Y),P(X))]. \end{array}$$

The optimality of the proposed criterion (6) is supported by Theorem 1.

**Theorem 1**. *The set of next actions* $A\in F_{L}^{m_{o}{\;}_{j}}$ *that maximizes the* $\text{IG }(z_{j};X_{j}^{\text{A}}|X_{j}^{m_{o}{\;}_{j}})$ *minimizes the expected KL divergence between the posterior distribution over* **z**_*j*_ *after all modality information has been observed and after* **A** *has been executed*.

$$\begin{array}{l} \underset{A\in F_{L}^{m_{o}{\;}_{j}}}{\text{argmin}}E_{X_{j}^{M\setminus m_{o}{\;}_{j}}|X_{j}^{m_{o}{\;}_{j}}}[\text{KL }(P(z_{j}|X_{j}^{M}),P(z_{j}|X_{j}^{m_{o}{\;}_{j}\cup A}))]\\ \;=\underset{A\in F_{L}^{m_{o}{\;}_{j}}}{\text{argmin}}\text{IG }(z_{j};X_{j}^{A}|X_{j}^{m_{o}{\;}_{j}}) \end{array}$$

*Proof*. See Appendix A.

This theorem is essentially the result of well-known characteristics of IG (see MacKay, 2003; Russo and Van Roy, 2016 for example). This means that maximizing IG is the optimal policy for active perception in an MHDP-based multimodal object category recognition task. As a special case, when only a single action is permitted, the following corollary is satisfied.

**Corollary** 1.1. *The next action m ∈ **M** \ m_**o***j*_ that maximizes $\text{IG }(z_{j};X_{j}^{m}|X_{j}^{m_{o}{\;}_{j}})$ minimizes the expected KL divergence between the posterior distribution over **z**_*j*_ after all modality information has been observed and after the action has been executed*.

$$\begin{array}{l} \underset{m\in M\setminus m_{o}{\;}_{j}}{\text{argmin}}E_{X_{j}^{M\setminus m_{o}{\;}_{j}}|X_{j}^{m_{o}{\;}_{j}}}[\text{KL }(P(z_{j}|X_{j}^{M}),P(z_{j}|X_{j}^{\{m\}\cup m_{o}{\;}_{j}}))]\\ \;=\underset{m\in M\setminus m_{o}{\;}_{j}}{\text{argmin}}\text{IG }(z_{j};X_{j}^{m}|X_{j}^{m_{o}{\;}_{j}}). \end{array}$$

*Proof*. By substituting {*m*} into **A** in Theorem 1, we can obtain the corollary.

Using IG, the active perception strategy for the next single action is simply described as follows:

(9) $$\begin{array}{l} m_{j}^{*}=\underset{m\in M\setminus m_{o}{\;}_{j}}{\text{argmin}}\text{ IG }(z_{j};X_{j}^{m}|X_{j}^{m_{o}{\;}_{j}}). \end{array}$$

This means that the robot should select the action $m_{j}^{*}$ that can obtain the $X_{j}^{m_{j}^{*}}$ that maximizes the IG for the recognition result **z**_*j*_ under the condition that the robot has already observed $X_{j}^{m_{o}{\;}_{j}}$.

However, we still have two problems, as follows.

1. The argmax operation in (6) is a combinatorial optimization problem and incurs heavy computational cost when #(**M** \ *m*_*o*_*j*__) and *L* become large.
2. The calculation of $\text{IG }(z_{j};X_{j}^{A}|X_{j}^{m_{o}{\;}_{j}})$ cannot be performed in a straightforward manner.

Based on some properties of the MHDP, we can obtain reasonable solutions for these two problems.

### 4.2. Sequential decision making as a submodular maximization

If a robot wants to select *L* actions **A**_*j*_ = {*a*_1_, *a*_2_, …, *a*_*L*_} (*a*_*i*_ ∈ **M** \ *m*_*o*_*j*__), it has to solve (6), i.e., a combinatorial optimization problem. The number of combinations of *L* actions is _#(**M** \ *m*_*o*_*j*__)_*C*_*L*_, which increases dramatically when the number of possible actions #(**M** \ *m*_*o*_*j*__) and *L* increase. For example, Sinapov et al. (2014) gave a robot 10 different behaviors in their experiment on robotic multimodal categorization. Future autonomous robots will have more available actions for interacting with a target object and be able to obtain additional types of modality information through these interactions. Hence, it is important to develop an efficient solution for the combinatorial optimization problem.

Here, the MHDP has advantages for solving this problem.

**Theorem 2**. *The evaluation criterion for multimodal active perception* $\text{IG }(z_{j};X_{j}^{A}|X_{j}^{m_{o}{\;}_{j}})$ *is a submodular and non-decreasing function with regard to* **A**.

*Proof*. As shown in the graphical model of the MHDP in Figure 2, the observations for each modality $X_{j}^{m}$ are conditionally independent under the condition that a set of latent variables $z_{j}=\{{\left\{k_{jt}\right\}}_{1\leq t\leq T_{j}},{\left\{t_{jn}^{m}\right\}}_{m\in M,1\leq n\leq N_{j}^{m}}\}$ is given. This satisfies the conditions of the theorem by Krause and Guestrin (2005). Therefore, $\text{IG }(z_{j};X_{j}^{m}|X_{j}^{m_{o}{\;}_{j}})$ is a submodular and non-decreasing function with regard to $X_{j}^{m}$.

Submodularity is a property similar to the convexity of a real-valued function in a vector space. If a set function *F* : *V* → *R* satisfies

$$\begin{array}{l} F(A\cup x)-F(A)\geq F(A^{\prime }\cup x)-F(A^{\prime }), \end{array}$$

where *V* is a finite set ∀*A* ⊂ *A*′ ⊆ *V* and *x* ∉ *A*, the set function *F* has submodularity and is called a submodular function.

Function IG is not always a submodular function. However, Krause et al. proved that IG(*U*; *A*) is submodular and non-decreasing with regard to *A* ⊆ *S* if all of the elements of *S* are conditionally independent under the condition that *U* is given. With this theorem, Krause and Guestrin (2005) solved the sensor allocation problem efficiently. Theorem 2 means that the problem (6) is reduced to a *submodular maximization problem*.

It is known that the greedy algorithm is an efficient strategy for the submodular maximization problem. Nemhauser et al. (1978) proved that the greedy algorithm can select a subset that is at most a constant factor (1−1/e) worse than the optimal set, if the evaluation function *F*(*A*) is submodular, non-decreasing, and *F*(∅) = 0, where *F*(·) is a set function, and *A* is a set. If the evaluation function is a submodular set function, a greedy algorithm is practically sufficient for selecting subsets in many cases. In sum, a greedy algorithm gives a near-optimal solution. However, the greedy algorithm is still inefficient because it requires an evaluation of all choices at each step of a sequential decision making process.

Minoux (1978) proposed lazy greedy algorithm to make the greedy algorithm more efficient for the submodular evaluation function. The lazy greedy algorithm can reduce the number of evaluations by using the characteristics of a submodular function.

### 4.3. Monte Carlo approximation of IG

Equations (6) and (9) provide a robot with an appropriate criterion for selecting an action to efficiently recognize a target object. However, at first glance, it looks difficult to calculate the IG. First, the calculation of the expectation procedure $E_{X_{j}^{A}|X_{j}^{m_{o}{\;}_{j}}}[\cdot ]$ requires a sum operation over all possible $X_{j}^{\text{A}}$. The number of possible $X_{j}^{\text{A}}$ exponentially increases when the number of elements in the BoF increases. Second, the calculation of $P(z_{j}|X_{j}^{A\cup m_{o}{\;}_{j}})$ for each possible observation $X_{j}^{\text{A}}$ requires the same computational cost as recognition in the multimodal categorization itself. Therefore, the straightforward calculation for solving (9) is computationally impossible in a practical sense.

However, by exploiting a characteristic property of the MHDP, a Monte Carlo approximation can be derived. First, we describe IG as the expectation of a logarithm term.

(10) $$\begin{array}{l} \text{IG }(z_{j};X_{j}^{m}|X_{j}^{m_{o}{\;}_{j}})\text{ ​}=\text{​}\sum_{z_{j},\;X_{j}^{m}}P(z_{j},X_{j}^{m}|X_{j}^{m_{o}{\;}_{j}})\log\frac{P(z_{j},X_{j}^{m}|X_{j}^{m_{o}{\;}_{j}})}{P(z_{j}|X_{j}^{m_{o}{\;}_{j}})P(X_{j}^{m}|X_{j}^{m_{o}{\;}_{j}})}\\ \;=E_{z_{j},\;X_{j}^{m}|X_{j}^{m_{o}{\;}_{j}}}[\log\frac{P(z_{j},X_{j}^{m}|X_{j}^{m_{o}{\;}_{j}})}{P(z_{j}|X_{j}^{m_{o}{\;}_{j}})P(X_{j}^{m}|X_{j}^{m_{o}{\;}_{j}})}]. \end{array}$$

An analytic evaluation of (10) is also practically impossible. Therefore, we adopt a Monte Carlo method. Equation (10) suggests that an efficient Monte Carlo approximation can be performed as shown below if we can sample

$$\begin{array}{l} (z_{j}^{\left[k\right]},X_{j}^{m[k]})\sim P(z_{j},X_{j}^{m}|X_{j}^{m_{o}{\;}_{j}}),\;(k\in \{1,\ldots ,K\}). \end{array}$$

Fortunately, the MHDP provides a sampling procedure for $z_{j}^{\left[k\right]}\sim P(z_{j}|X_{j}^{m_{o}{\;}_{j}})$ and $X_{j}^{m\left[k\right]}\sim P\left(X_{j}^{m}|z_{j}^{\left[k\right]}\right)$ in its original paper (Nakamura et al., 2011b). In the context of multimodal categorization by a robot, $X_{j}^{m\left[k\right]}\sim P\left(X_{j}^{m}|z_{j}^{\left[k\right]}\right)$ is a prediction of an unobserved modality's sensation using observed modalities' sensations, i.e., cross-modal inference. The sampling process of $\left(z_{j}^{\left[k\right]},X_{j}^{m\left[k\right]}\right)$ can be regarded as a mental simulation by a robot that predicts the unobserved modality's sensation leading to a categorization result based on the predicted sensation and observed information.

(11) $$\begin{array}{l} (10)\approx \frac{1}{K}\sum_{k}\log\frac{P(z_{j}^{\left[k\right]},X_{j}^{m[k]}|X_{j}^{m_{o}{\;}_{j}})}{P(z_{j}^{\left[k\right]}|X_{j}^{m_{o}{\;}_{j}})P(X_{j}^{m[k]}|X_{j}^{m_{o}{\;}_{j}})}\\ \;=\frac{1}{K}\sum_{k}\log\frac{P(X_{j}^{m[k]}|z_{j}^{\left[k\right]},X_{j}^{m_{o}{\;}_{j}})}{\underset{*}{\underset{︸}{P(X_{j}^{m[k]}|X_{j}^{m_{o}{\;}_{j}})}}}. \end{array}$$

In (11), $P(X_{j}^{m[k]}|z_{j}^{\left[k\right]},X_{j}^{m_{o}{\;}_{j}})$ in the numerator can be easily calculated because all the parent nodes of $X_{j}^{m\left[k\right]}$ are given in the graphical model shown in Figure 2. However, $P(X_{j}^{m[k]}|X_{j}^{m_{o}{\;}_{j}})$ in the denominator cannot be evaluated in a straightforward way. Again, a Monte Carlo method can be adopted, as follows:

(12) $$\begin{array}{l} \left(*)=P(X_{j}^{m[k]}|X_{j}^{m_{o}{\;}_{j}})=\sum_{z_{j}}P(X_{j}^{m[k]}|z_{j},X_{j}^{m_{o}{\;}_{j}})P(z_{j}|X_{j}^{m_{o}{\;}_{j}}\right)\\ \;=E_{z_{j}|X_{j}^{m_{o}{\;}_{j}}}[P(X_{j}^{m[k]}|z_{j},X_{j}^{m_{o}{\;}_{j}})]\\ \;\approx \frac{1}{K^{\prime }}\sum_{k^{\prime }}P(X_{j}^{m[k]}|z_{j}^{\left[k^{\prime }\right]},X_{j}^{m_{o}{\;}_{j}}) \end{array}$$

where *K*′ is the number of samples for the second Monte Carlo approximation. Fortunately, in this Monte Carlo approximation (12), we can reuse the samples drawn in the previous Monte Carlo approximation efficiently, i.e., *K*′ = *K*. By substituting (12) for (11), we finally obtain the approximate IG for the criterion of active perception, i.e., our proposed method, as follows:

$$\begin{array}{l} \text{IG }(z_{j};X_{j}^{m}|X_{j}^{m_{o}{\;}_{j}})\approx \frac{1}{K}\sum_{k}\log\frac{P(X_{j}^{m[k]}|z_{j}^{\left[k\right]},X_{j}^{m_{o}{\;}_{j}})}{\frac{1}{K}\sum_{k^{\prime }}P(X_{j}^{m[k]}|z_{j}^{\left[k^{\prime }\right]},X_{j}^{m_{o}{\;}_{j}})}. \end{array}$$

Note that the computational cost for evaluating IG becomes *O*(*K*^2^). In summary, a robot can approximately estimate the IG for unobserved modality information by generating virtual observations based on observed data and evaluating their likelihood.

### 4.4. MHDP-based active perception methods

We propose the use of the *greedy* and *lazy greedy algorithms* for selecting *L* actions to recognize a target object on the basis of the submodular property of IG. The final greedy and lazy greedy algorithms for MHDP-based active perception, i.e., our proposed methods, are shown in Algorithms 1 and 2, respectively.

**Algorithm 1:** Greedy algorithm.

**Require:** MHDP is trained using a training data set.
The *j*-th object is found.
**m**_**o***j*_ is initialized, and $X_{j}^{m_{\text{o}j}}$ is observed.
**for** *l* = 1 to *L* **do**
**for all** *m* ∈ **M** \ **m**_**o***j*_ **do**
**for** *k* = 1 to *K* **do**
Draw $$(z_{j}^{\left[k\right]},X_{j}^{m[k]})\sim P\left(zj,X_{j}^{m}|X_{j}^{{\text{m}}_{\text{o}j}}\right)$$
**end for**
$$IG_{m}\leftarrow \frac{1}{K}\sum_{k}\log\frac{P(X_{j}^{m[k]}|{\text{z}}_{j}^{\left[k\right]},X_{j}^{{\text{m}}_{\text{o}j}})}{\frac{1}{K}\underset{k^{\prime }}{\sum }P(X_{j}^{m[k]}|{\text{z}}_{j}^{\left[k^{\prime }\right]},X_{j}^{{\text{m}}_{\text{o}j}})}$$
**end for**
$$m^{*}\leftarrow \underset{m}{\mathrm{argmax}}\text{I}{\text{G}}_{m}$$
Execute the m^*^-th action to the *j*-th target object and obtain $X_{j}^{m^{*}}$.
**m**_**o***j*_ ← **m**_**o***j*_ ∪ {m^*^}
**end for**

**Algorithm 2:** Lazy greedy algorithm.

**Require:** The MHDP is trained using a training data set.
The *j*-th object is found.
***m***_***o**j*_ is initialized, and $X_{j}^{m_{o}{}_{j}}$ is observed.
**for all** *m* ∈ **M** \ _***m***_***o***_*j*_ **do**
**for** *k* = 1 to *K* **do**
Draw $$\left(z_{j}^{\left[k\right]},X_{j}^{m[k]})\sim P(z_{j},X_{j}^{m}|X_{j}^{m_{o}{}_{j}}\right)$$
**end for**
$${\text{IG}}_{m}\leftarrow \frac{1}{K}\sum_{k}\log\frac{P(X_{j}^{m[k]}|z_{j}^{\left[k\right]},X_{j}^{m_{o}{}_{j}})}{\frac{1}{K}\sum_{k^{\prime }}P\left(X_{j}^{m[k]}|z_{j}^{\left[k^{\prime }\right]},X_{j}^{m_{o}{}_{j}}\right)}$$
**end for**
$$m^{*}\leftarrow \underset{m}{\arg\max}{\text{ IG}}_{m}$$
Execute the m^*^-th action to the *j*-th target object and obtain $X_{j}^{m^{*}}$.
**m**_**o**_*j*__ ← **m**_**o**_*j*__ ∪ {m^*^}
Prepare a stack *S* for the modality indices and initialize it.
**for all** *m*∈**M**\_*m*_*o*_*j*_ **do**
*push*(*S*, (*m*, IG_*m*_))
**end for**
**for** *l* = 1 to *L*−1 **do**
**repeat**
*S*←*descending*_*sort*(*S*) // w.r.t. IG_*m*_
$\left(m^{1},\text{I}{\text{G}}_{m^{1}}\right)\leftarrow pop\left(S\right)$ , $\left(m^{2},\text{I}{\text{G}}_{m^{2}}\right)\leftarrow pop\left(S\right)$
// Re-evaluate $\text{I}{\text{G}}_{m^{1}}$ as follows.
**for** *k* = 1 to *K* **do**
Draw $$\left(z_{j}^{\left[k\right]},X_{j}^{m^{1}[k]})\sim P(z_{j},X_{j}^{m^{1}}|X_{j}^{m_{o}{}_{j}}\right)$$
**end for**
$${\text{IG}}_{m^{1}}\leftarrow \frac{1}{K}\sum_{k}\log\frac{P(X_{j}^{m^{1}[k]}|z_{j}^{\left[k\right]},X_{j}^{m_{o}{}_{j}})}{\frac{1}{K}\sum_{k^{\prime }}P\left(X_{j}^{m^{1}[k]}|z_{j}^{\left[k^{\prime }\right]},X_{j}^{m_{o}{}_{j}}\right)}$$
*push*(*S*, (*m*^2^, IG_*m*^2^_)), *push*(*S*, (*m*^1^, IG_*m*^1^_))
**until** IG_*m*^1^_ ≥ IG_*m*^2^_
m^*^ ← *m*^1^
*pop*(*S*)
Execute the m^*^-th action to the *j*-th target object and obtain $X_{j}^{m^{*}}$.
**m**_**o**_*j*__ ← **m**_**o**_*j*__ ∪ {m^*^}
**end for**

The main contribution of the lazy greedy algorithm is to reduce the computational cost of active perception. The majority of the computational cost originates from the number of times a robot evaluates IG_*m*_ for determining action sequences. When a robot has to choose *L* actions, the brute-force algorithm that directly evaluates all alternatives $A\in F_{L}^{m_{o}{\;}_{j}}$ using (6) requires _#(**M** \ *m*_*o*_*j*__)_*C*_*L*_ evaluations of $\text{IG }(z_{j};X_{j}^{A}|X_{j}^{m_{o}{\;}_{j}})$. In contrast, the greedy algorithm requires {#(**M** \ *m*_*o*_*j*__) + (#(**M** \ *m*_*o*_*j*__)−1) + … + (#(**M** \ *m*_*o*_*j*__)−*L* + 1)} evaluations of $\text{IG }(z_{j};X_{j}^{m}|X_{j}^{m_{o}{\;}_{j}})$, i.e., *O*(*ML*). The lazy greedy algorithm incurs the same computational cost as the greedy algorithm only in the worst case. However, practically, the number of re-evaluations in the lazy greedy algorithm is quite small. Therefore, the computational cost of the lazy greedy algorithm increases almost in proportion to *L*, i.e., almost linearly. The memory requirement of the proposed method is also quite small. Both the greedy and lazy greedy algorithms only require memory for IG_*m*_ for each modality and *K* samples for the Monte Carlo approximation. These requirements are negligibly small compared with the MHDP itself.

Note that the IG_*m*_ is not the exact IG, but an approximation. Therefore, the differences between IG and IG_*m*_ may harm the performance of greedy and lazy greedy algorithms to a certain extent. However, the algorithms are expected to work practically. We evaluated the algorithms through experiments.

## 5. Experiment 1: humanoid robot

### 5.1. Conditions

**Figure 3:**
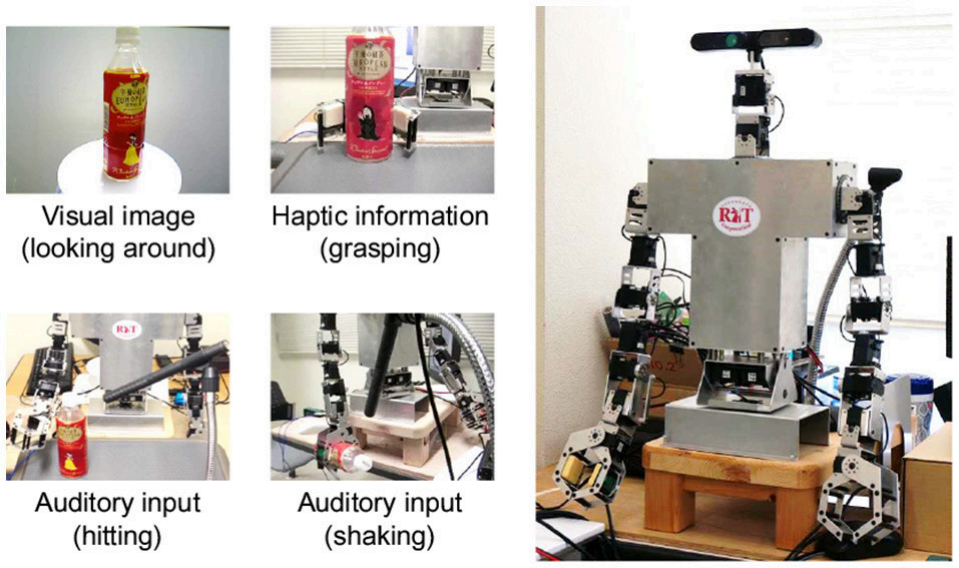
A humanoid robot used in the experiment.

An experiment using an upper-torso humanoid robot was conducted to verify the proposed active perception method in the real-world environment. In this experiment, RIC-Torso, developed by the RT Corporation, was used (see Figure 3). RIC-Torso is an upper-torso humanoid robot that has two robot hands. We prepared an experimental environment that is similar to the one in the original MHDP paper (Nakamura et al., 2011b). The robot has four available actions and four corresponding modality information. The set of modalities was **M** = {*m*^*v*^, *m*^*as*^, *m*^*ah*^, *m*^*h*^}, which represent visual information, auditory information obtained by shaking an object, one by hitting an object and haptic information, respectively.

#### 5.1.1. Visual information (*m*^*v*^)

Visual information was obtained from the Xtion PRO LIVE set on the head of the robot. The camera was regarded as the eyes of the robot. The robot captured 74 images of a target object while it rotated on a turntable (see Figure 3). The size of each image was re-sized to 320 × 240. Scale-invariant feature transform (SIFT) feature vectors were extracted from each captured image (Lowe, 2004). A certain number of 128-dimensional feature vectors were obtained from each image. Note that the SIFT feature did not consider hue information. All of the obtained feature vectors were transformed into BoF representations using k-means clustering with *k* = 25. The number of clusters *k* was determined empirically, considering prior works (Nakamura et al., 2011b; Araki et al., 2012). The k-means clustering was performed using data from all objects in a training set, and the centroids of the clusters were determined. BoF representations were used as observation data for the visual modality of the MHDP. The index for this modality was defined as *m*^*v*^.

#### 5.1.2. Auditory information (*m*^*as*^ and *m*^*ah*^)

Auditory information was obtained from a multipowered shotgun microphone NTG-2 by RODE Microphone. The microphone was regarded as the ear of the robot. In this experiment, two types of auditory information were acquired. One was generated by hitting the object, and the other was generated by shaking it. The two sounds were regarded as different auditory information and hence different modality observations in the MHDP model. The two actions, i.e., hitting and shaking, were manually programmed for the robot. Each action was implemented as a fixed trajectory. When the robot began to execute an action, it also started recording the objects's sound (see Figure 3). The sound was recorded until two seconds after the robot finished the action. The recorded auditory data were temporally divided into frames, and each frame was transformed into 13-dimensional Mel-frequency cepstral coefficients (MFCCs). The MFCC feature vectors were transformed into BoF representations using k-means clustering with *k* = 25 in the same way as the visual information. The indices of these modalities were defined as *m*^*as*^ and *m*^*ah*^, respectively, for “shake” and “hit.”

#### 5.1.3. Haptic information (*m*^*h*^)

Haptic information was obtained by grasping a target object using the robot's hand. When the robot attempted to obtain haptic information from an object placed in front of it, it moved its hand to the object and gradually closed its hand until a certain amount of counterforce was detected (see Figure 3). The joint angle of the hand was measured when the hand touched the target object and when the hand stopped. The two variables and difference between the two angles were used as a three-dimensional feature vector. When obtaining haptic information, the robot grasped the target object 10 times and obtained 10 feature vectors. The feature vectors were transformed into BoF representations using k-means clustering with *k* = 5 in the same way as for the other information types. The index of the haptic modality was defined as *m*^*h*^.

#### 5.1.4. Multimodal information as BoF representations

In summary, a robot could obtain multimodal information from four modalities for perception. The dimensions of the BoFs were set to 25, 25, 25, and 5 for *m*^*v*^, *m*^*as*^, *m*^*ah*^, and *m*^*h*^, respectively. The dimension of each BoF corresponds to the number of clusters for k-means clustering. The numbers of clusters, i.e., the sizes of the dictionaries, were empirically determined on the basis of a preliminary experiment on multimodal categorization. All of the training datasets were used to train the dictionaries. The histograms of the feature vectors, i.e., the BoFs, were resampled to make their counts $N_{j}^{m^{v}}=100,N_{j}^{m^{as}}=80,N_{j}^{m^{ah}}=130$, and $N_{j}^{m^{h}}=30$. The weight of each modality *w*^*m*^ was set to 1. The formation of multimodal object categories itself is out of the scope of this paper. Therefore, the constants were empirically determined so that the robot could form object categories that are similar to human participants. The number of samples *K* in the Monte Carlo approximation for estimating IG was set to *K* = 5, 000. The constant *K* was determined empirically. The effect of *K* will be examined in the experiment as well (see **Figure 11**).

#### 5.1.5. Target objects

**Figure 4:**
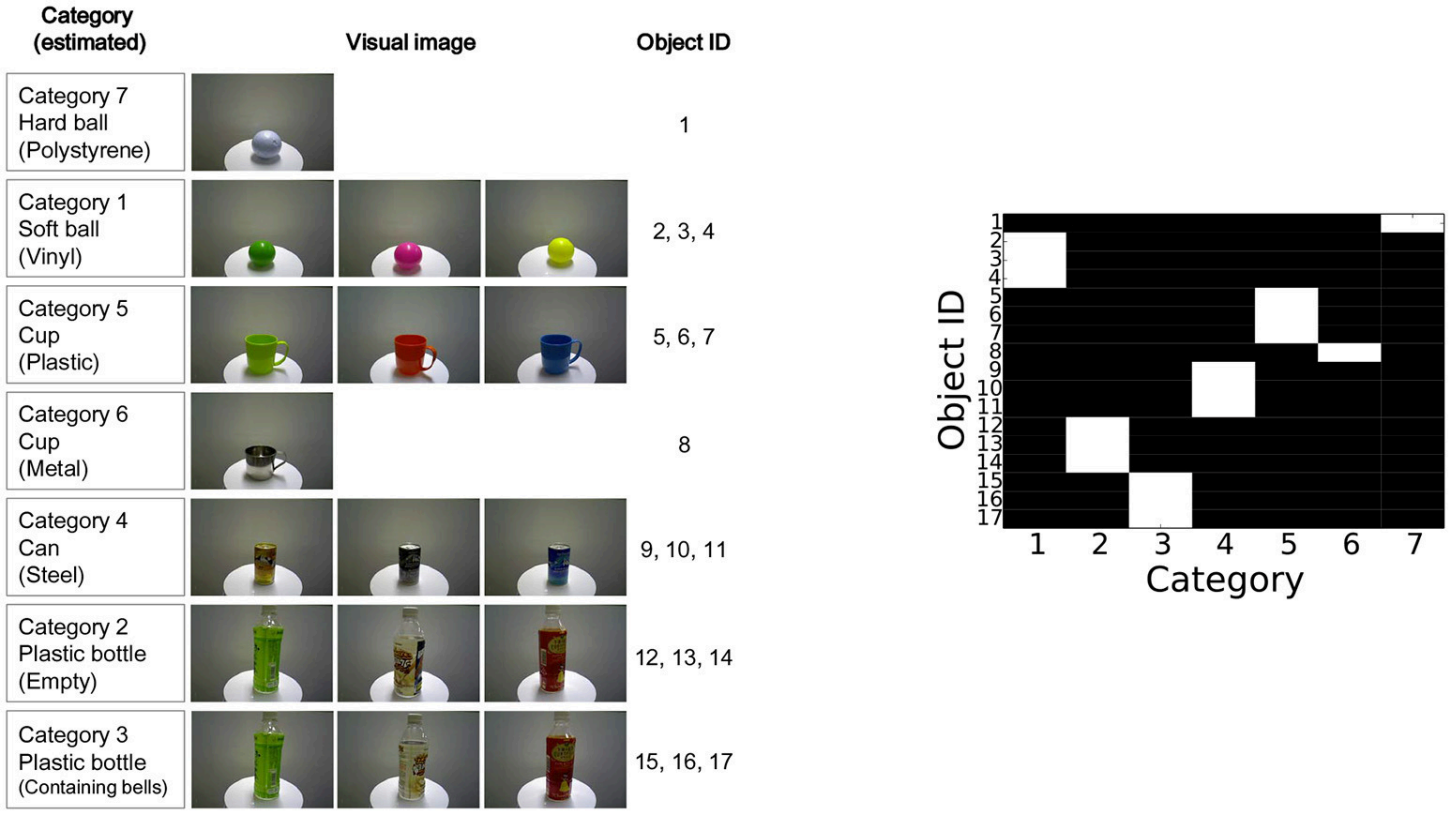
**(Left)** target objects used in the experiment and **(Right)** categorization results obtained in the experiment.

For the target objects, 17 types of commodities were prepared for the experiment shown in Figure 4. An object was provided for obtaining a training data, i.e., data for object categorization, and another object was provided for obtaining test data, i.e., data for active perception, for each type of objects. Each index on the right-hand side of the figure indicates the index of each object. The hardness of the balls, the striking sounds of the cups, and the sounds made while shaking the bottles were different depending on the object categories. Therefore, ground-truth categorization could not be achieved using visual information alone.

### 5.2. Procedure

The experimental procedure was as follows. First, the robot formed object categories through multimodal categorization in an unsupervised manner. An experimenter placed each object in front of the robot one by one. In this training phase, two objects for each type of objects were provided. The robot looked at the object to obtain visual features, grasped it to obtain haptic features, shook it to obtain auditory shaking features, and hit it to obtain the auditory striking features. After obtaining the multimodal information of the objects as a training data set, the MHDP was trained using a Gibbs sampler. The results of multimodal categorization are shown in Figure 4. The category that has the highest posterior probability for each object is shown in white. These results show that the robot can form multimodal object categories using MHDP, as described in Nakamura et al. (2011b). After the robot had formed object categories, we fixed the latent variables for the training data set^3^.

Second, an experimental procedure for active perception was conducted. An experimenter placed an object in front of the robot. The robot observed the object using its camera, obtained visual information, and set $m_{o}{\;}_{j}=\{m^{v}\}$. An object was provided for each type of objects shown in Figure 4 to the robot one by one. Therefore, 17 objects were used for evaluating each active perception strategy. The sequential action selection and object recognition were performed once per an object. At each step of the sequential action selection, Gibbs sampler for MHDP was performed and it updated its latent variables, i.e., recognition state, of the MHDP. The robot then determined its next set of actions for recognizing the target object using its active perception strategy shown in Algorithms 1 and 2.

### 5.3. Results

#### 5.3.1. Selecting the next action

First, we describe results for the first single action selection after obtaining visual information. In this experiment, the robot had three choices for its next action, i.e., *m*^*as*^, *m*^*ah*^, and *m*^*h*^. To evaluate the results of active perception, we used $\text{KL }(P(k|X_{j}^{M}),P(k|X_{j}^{A\cup m_{o}{\;}_{j}}))$, i.e., the distance between the posterior distribution over the object categories *k* in the final recognition state and that in the next recognition state as an evaluation criterion on behalf of $\text{KL }(P(z_{j}|X_{j}^{M}),P(z_{j}|X_{j}^{A\cup m_{o}{\;}_{j}}))$, which is the original evaluation criterion in (4). The computational cost for numerical evaluation of $\text{KL }(P(z_{j}|X_{j}^{M}),P(z_{j}|X_{j}^{A\cup m_{o}{\;}_{j}}))$ using a Monte Carlo method is too high because $z_{j}=\{{\left\{k_{jt}\right\}}_{1\leq t\leq T_{j}},{\left\{t_{jn}^{m}\right\}}_{m\in M,1\leq n\leq N_{j}^{m}}\}$ has so many variables and a posterior distributions over **z**_*j*_ is very complex.

**Figure 5:**
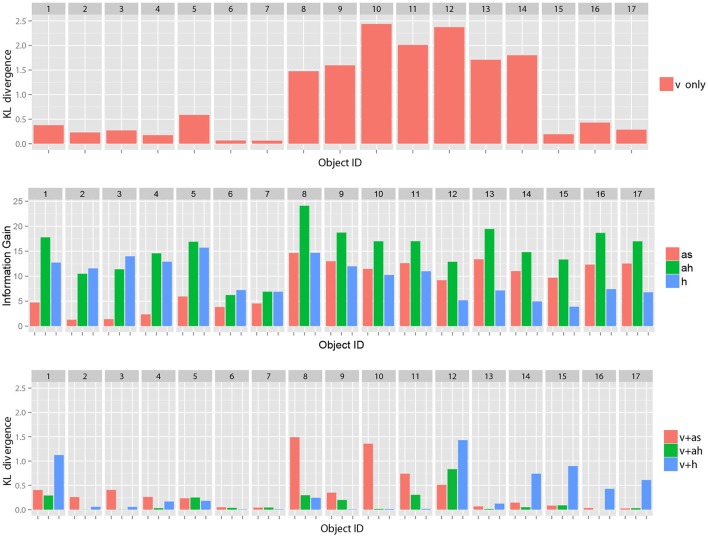
**(Top)** Samples of KL divergence between the final recognition state and the posterior probability estimated after obtaining only visual information, **(Middle)** samples of estimated IG_*m*_ for each object based on visual information (v), and **(Bottom)** samples of KL divergence between the final recognition state and the posterior probability estimated after obtaining only visual information and each selected action where as, ah, h represent represent auditory information obtained by shaking an object, one by hitting an object and haptic information, respectively. Our theory of multimodal active perception suggests that the action with the highest information gain (shown in the middle) tends to lead its initial recognition state (whose KL divergence from the final recognition state is shown at the top) to a recognition state whose KL divergence from the final recognition state (shown at the bottom) is the smallest. These figures suggest the probabilistic relationships were satisfied as a whole.

Figure 5 (Top) shows samples of the KL divergence between the posterior probabilities of the category after obtaining the information from all modalities and after obtaining only visual information.

**Figure 6:**
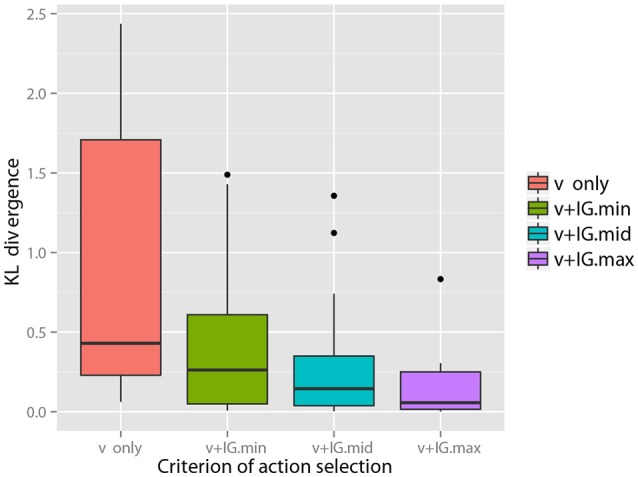
Reduction in the KL divergence by executing an action selected on the basis of the IG_*m*_ maximization criterion. The KL divergences between the recognition state after executing the second action and the final recognition state are calculated for all objects and shown with box plot. This shows that an action with more information brings the recognition of its state closer to the final recognition state.

With regard to some objects, e.g., objects 6 and 7, the figure shows samples of that visual information seems to be sufficient for the robot to recognize the objects as compared the other objects^4^. However, with regard to many objects, visual information alone could not lead the recognition state to the final state. However, it could be reached using the information of all modalities. Figure 5 (Middle) shows samples of IG_*m*_ calculated using the visual information for each action. Figure 5 (Bottom) shows the KL divergence between the final recognition state and the posterior probability estimated after obtaining visual information and the information of each selected action. We observe that an action with a higher value of IG_*m*_ tended to further reduce the KL divergence, as Theorem 1 suggests. Figure 6 shows the average KL divergence for the final recognition state after executing an action selected by the IG_*m*_ criterion. Actions IG.min, IG.mid, and IG.max denote actions that have the minimum, middle, and maximum values of IG_*m*_, respectively. These results show that IG.max clearly reduced the uncertainty of the target objects.

The precision of category recognition after an action execution is summarized in Table 1. Basically, a category recognition result is obtained as the posterior distribution (3) in the MHDP. The category with the highest posterior probability is considered to be the recognition result for illustrative purposes in Table 1. Obtaining information by executing IG.max almost always increased recognition performance.

**Table 1:** Number of successfully recognized objects.

**v only**	**v+IG.min**	**v+IG.mid**	**v+IG.max**	**Full information**
8/17	11/17	15/17	**1**6/17	17/17

**Figure 7:**
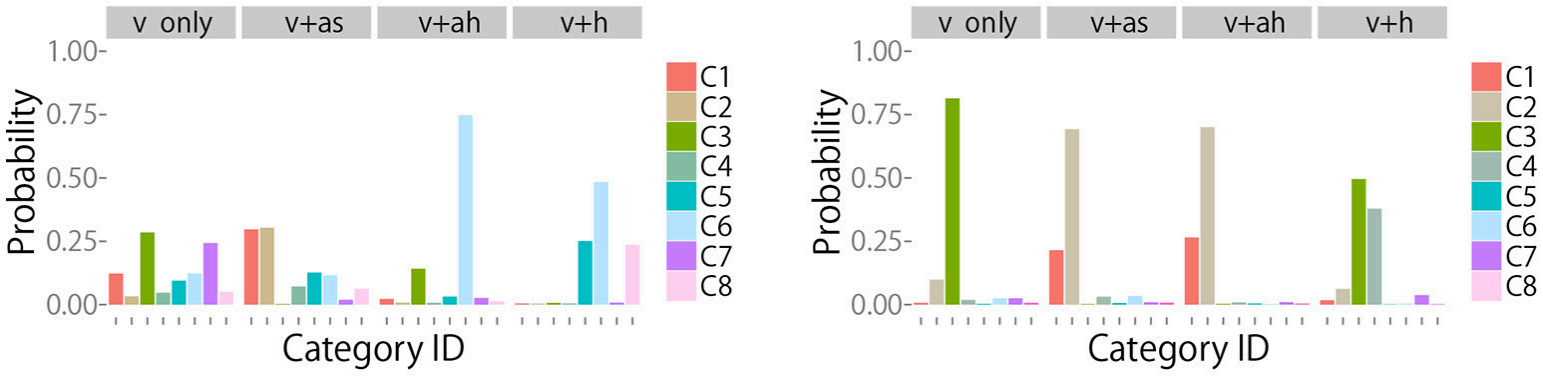
**(Left)** Posterior probability of the category for object 8 after executing each action. These results show that the action with the highest information gain, i.e., *ah*, allowed the robot to efficiently estimate that the true object category was “metal cup”. **(Right)** Posterior probability of the category for object 12 after executing each action. These results show that the actions with the highest and second highest information gain, i.e., *ah* and *as*, allowed the robot to efficiently estimate that the true object category was “empty plastic bottle”.

Examples of changes in the posterior distribution are shown in Figure 7 (Left, Right) for objects 8 (“metal cup”) and 12 (“empty plastic bottle”), respectively. The robot could not clearly recognize the category of object 8 after obtaining visual information. Action IG_*m*_ in Figure 5 shows that *m*^*ah*^ was IG.max for the 8th object. Figure 7 (Left) shows that *m*^*ah*^ reduced the uncertainty and allowed the robot to correctly recognize the object, as evidenced by category 6, a metal cup. This means that the robot noticed that the target object was a metal cup by hitting it and listening to its metallic sound. The metal cup did not make a sound when the robot shook it. Therefore, the IG for *m*^*as*^ was small. As Figure 7 (Right) shows, the robot first recognized the 12th object as a plastic bottle containing bells with high probability and as an empty plastic bottle with a low probability. Figure 5 shows that the IG_*m*_ criterion suggested *m*^*ah*^ as the first alternative and *m*^*as*^ as the second alternative. Figure 7 (Right) shows that *m*^*as*^ and *m*^*ah*^ could determine that the target object was an empty plastic bottle, but *m*^*h*^ could not.

As humans, we would expect to differentiate an empty bottle from a bottle containing bells by shaking or hitting the bottle, and differentiate a metal cup from a plastic cup by hitting it. The proposed active perception method constructively reproduced this behavior in a robotic system using an unsupervised multimodal machine learning approach.

#### 5.3.2. Selecting the next set of multiple actions

**Figure 8:**
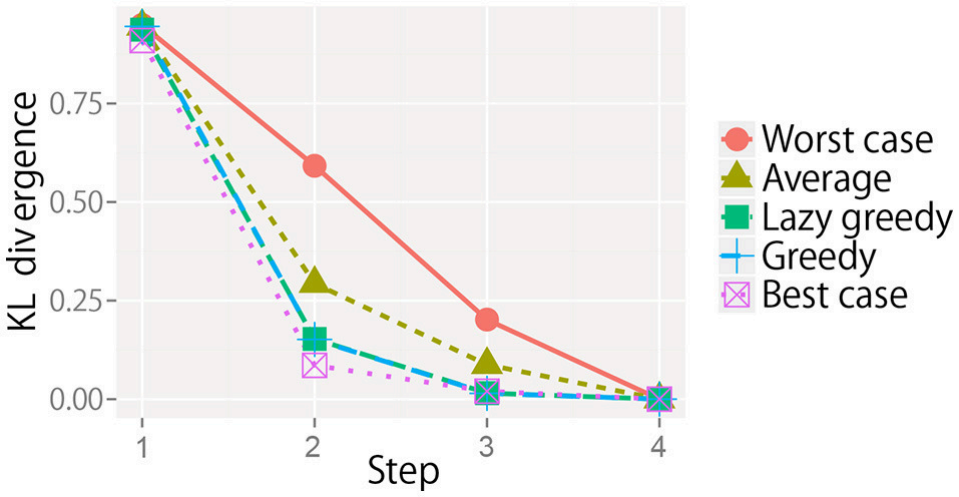
KL divergence from the final state at each step for each sequential action selection procedure. Note that the line of the lazy greedy algorithm is overlapped by that of the greedy algorithm.

We evaluated the greedy and lazy greedy algorithms for active perception sequential decision making. The KL divergence from the final state for all target objects is averaged at each step and shown in Figure 8. For each condition, the KL divergence gradually decreased and reached almost zero. However, the rate of decrease notably differed. As the theory of submodular optimization suggests, the greedy algorithm was shown to be a better solution on average and slightly worse than the best case (Nemhauser et al., 1978). The best and worst cases were selected after all types of sequential actions had been performed. The “average” is the average of the KL divergence obtained by all possible types of sequential actions. The results for the lazy greedy algorithm were almost same as those of the greedy algorithm, as Minoux (1978) suggested.

**Figure 9:**
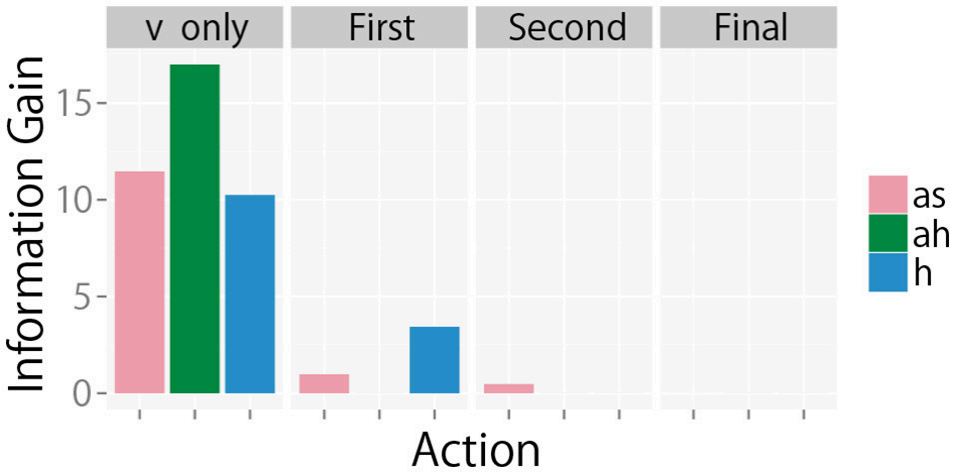
IG_*m*_ at each step for object 10 when the greedy algorithm is used.

The sequential behaviors of IG_*m*_ were observed to determine if their behaviors were consistent with our theories. For example, the changes in IG_*m*_ at each step as the robot sequentially selected its action to perform on object 10 using the greedy algorithm is shown in Figure 9. Theorem 2 shows that the IG is a submodular function. This predicts that IG_*m*_ decreases monotonically when a new action is executed in active perception. When the robot obtained only visual information (v only in Figure 9), all values of IG_*m*_ were still large. After *m*^*ah*^ was executed on the basis of the greedy algorithm, ${\mathrm{IG}}_{m^{ah}}$ became zero. At the same time, ${\mathrm{IG}}_{m^{as}}$ and ${\mathrm{IG}}_{m^{h}}$ decreased. In the same way, all values of IG_*m*_ gradually decreased monotonically.

**Figure 10:**
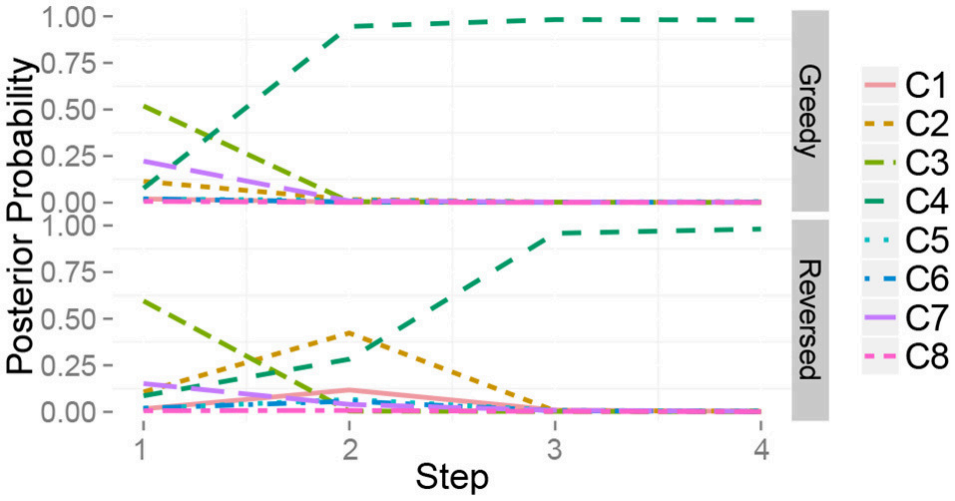
Time series of the posterior probability of the category for object 10 during sequential action selection based on **(top)** the greedy algorithm, i.e., *m*^*ah*^ → *m*^*h*^ → *m*^*as*^, and **(bottom)** its reverse order, i.e., *m*^*as*^ → *m*^*h*^ → *m*^*ah*^.

Figure 10 shows the time series of the posterior probability of the category for object 10 during sequential active perception. Using only visual information, the robot misclassified the target object as a plastic bottle containing bells (category 3). The action sequence in reverse order did not allow the robot to recognize the object as a steel can at its first step and change its recognition state to an empty plastic bottle (category 4). After the second action, i.e., grasping (*m*^*h*^), the robot recognized the object as a steel can. In contrast, the greedy algorithm could determine that the target object was in category 4, i.e., steel can, with its first action.

**Figure 11:**
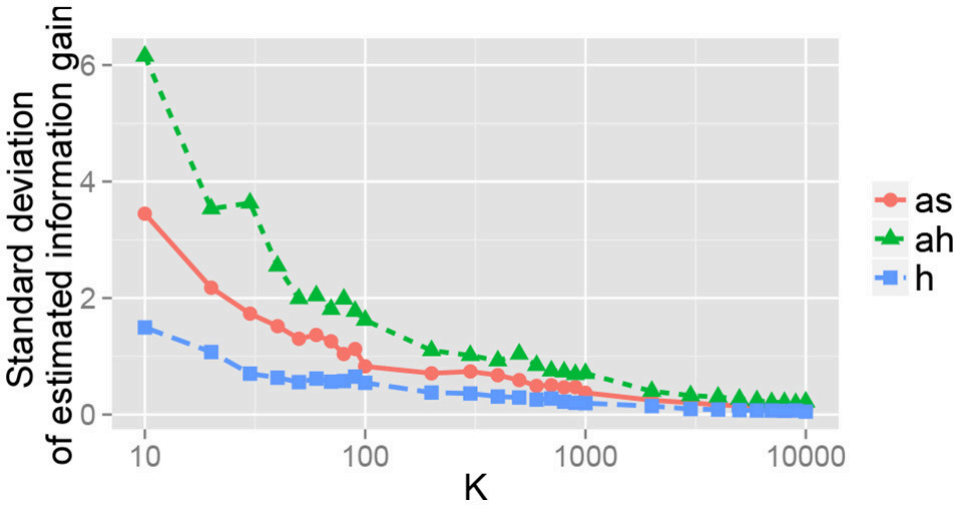
Standard deviation of the estimated information gain IG_*m*_ for the 15th object. For each *K*, 100 values of the estimated information gain IG_*m*_ were obtained, and their standard deviation is shown.

The effect of the number of samples *K* for the Monte Carlo approximation was observed. Figure 11 shows the relation between *K* and the standard deviation of the estimated IG_*m*_ for the 15th object for each action after obtaining a visual image. This figure shows that estimation error gradually decreases when *K* increases. Roughly speaking, *K* ≥ 1, 000 seems to be required for an appropriate estimate of IG_*m*_ in our experimental setting. Evaluation of IG_*m*_ required less than 1 second, which is far shorter than the time required for action execution by a robot. This means that our method can be used in a real-time manner.

These empirical results show that the proposed method for active perception allowed a robot to select appropriate actions sequentially to recognize an object in the real-world environment and in a real-time manner. It was shown that the theoretical results were supported, even in the real-world environment.

## 6. Experiment 2: synthetic data

In experiment 1, the numbers of classes, actions, and modalities as well as the size of dataset were limited. In addition, it was difficult to control the robotic experimental settings so as to check some interesting theoretical properties of our proposed method. Therefore, we performed a supplemental experiment, Experiment 2, using synthetic data comprising 21 object types, 63 objects, and 20 actions, i.e., modalities.

First, we checked the validity of our active perception method when the number of types of actions increases. Second, we checked how the method worked when two classes were assigned to the same object. Although the MHDP can categorize an object into two or more categories in a probabilistic manner, each object was classified into a single category in the previous experiment.

### 6.1. Conditions

A synthetic dataset was generated using the generative model that the MHDP assumes (see Figure 2). We prepared 21 virtual object classes, and three objects were generated from each object class, i.e., we obtained 63 objects in total. Among the object classes, 14 object classes are “pure,” and seven object classes are “mixed.” For each pure object class, a multinomial distribution was drawn from the Dirichlet distribution corresponding to each modality. We set the number of modalities *M* = 20. The hyperparameters of the Dirichlet distributions of the modalities were set to $\alpha_{0}^{m}=0.4\left(m-1\right)$ for *m* > 1. For *m* = 1, we set $\alpha_{0}^{1}=10$. For each mixed object class, a multinomial distribution for each modality was prepared by mixing the distributions of the two pure object classes. Specifically, the multinomial distribution for the *i*-th mixed object was obtained by averaging those of the (2*i*−1)-th and the 2*i*-th object classes. The observations for each modality of each object were drawn from the multinomial distributions corresponding to the object's class. The count of the BoFs for each modality was set to 20. Finally, 42 pure virtual objects and 21 mixed virtual objects were generated.

**Figure 12:**
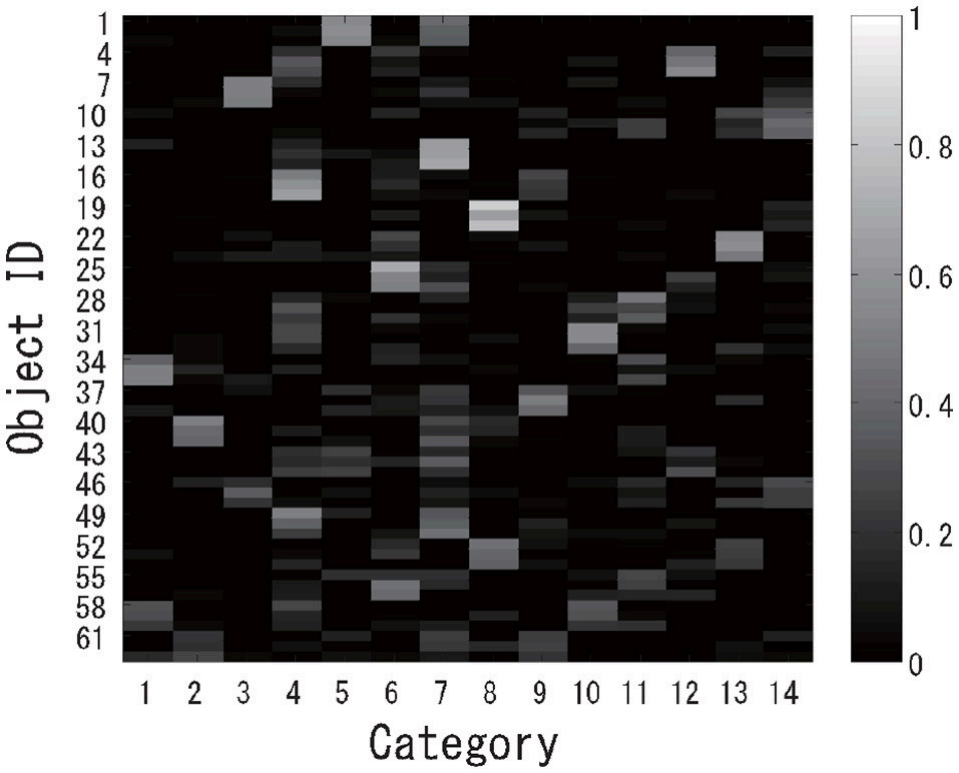
Categorization results for the posterior probability distributions for each object.

The experiment was performed almost in the same way as experiment 1. First, multimodal categorization was performed for the 63 virtual objects, and 14 categories were successfully formed in an unsupervised manner. The posterior distributions over the object categories are shown in Figure 12. Generally speaking, mixed objects were categorized into two or more classes. After categorization, a virtual robot was asked to recognize all of the target objects using the proposed active perception method.

### 6.2. Results

We compared the greedy, lazy greedy, and random algorithms for the active perception sequential decision making process. The random algorithm is a baseline method that determines the next action randomly from the remaining actions that have not been taken. In other words, the random algorithm is the case in which a robot does not employ any active perception algorithms.

**Figure 13:**
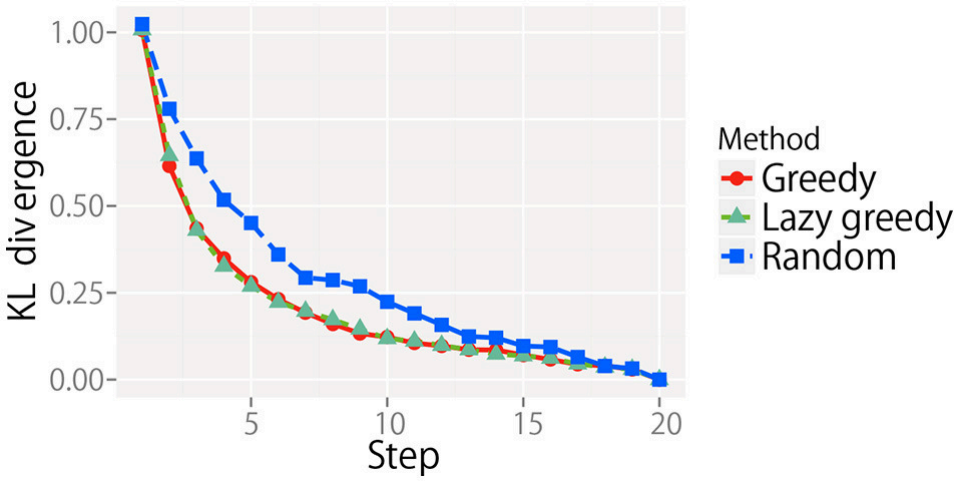
KL divergence from the final state at each step for each sequential action selection procedure.

The KL divergence from the final state for all target objects is averaged at each step and shown in Figure 13. For each condition, the KL divergence gradually decreased and reached almost zero. However, the rate of decrease was different. The greedy and lazy greedy algorithms were clearly shown to be better solutions on average than the random algorithm. In contrast with experiment 1, the best and worst cases could not practically be calculated because of the prohibitive computational cost. Interestingly, the lazy greedy algorithm has almost the same performance as the greedy algorithm, as the theory suggests, although the laziness reduced the computational cost in reality.

The number of times the robot evaluated IG_*m*_ to determine the action sequences for all executable counts of actions *L* = 1, 2, …, *M* is summarized for each method. The number of times the lazy greedy algorithm was required for each target object was 71.7 (*SD* = 5.2) on average, and that of the greedy algorithm was 190. Theoretically, the greedy and lazy greedy algorithms require *O*(*M*^2^) evaluations. Practically, the number of re-evaluations needed by the lazy greedy algorithm is quite small. In contrast, the brute-force algorithm requires *O*(2^*M*^) evaluations, i.e., far more evaluations of IG are required.

**Figure 14:**
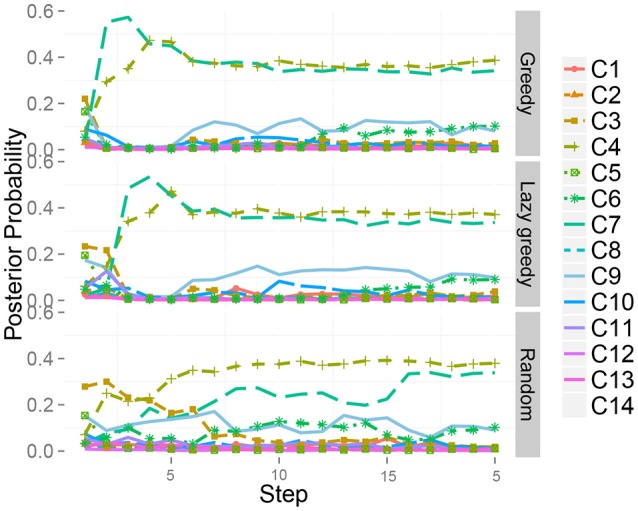
Time series of the posterior probability of the category for object 51 during sequential action selection based on **(Top)** the greedy algorithm, **(Middle)** the lazy greedy algorithm, and **(Bottom)** the random selection procedure.

Next, a case in which two classes were assigned to the same object was investigated. The target dataset contained “mixed” objects. The results also imply that our method works well even when two classes are assigned to the same object. This is because our theory is completely derived on the basis of the probabilistic generative model, i.e., the MHDP. We show a typical result. Figure 14 shows the time series of the posterior probability of the category for object 51, i.e., one of the mixed objects, during sequential active perception. This shows that the greedy and lazy greedy algorithms quickly categorized the target object into two categories “correctly.” Our formulation assumes the categorization result to be a posterior distribution. Therefore, this type of probabilistic case can be treated naturally.

## 7. Conclusion and discussion

In this paper, we described an MHDP-based active perception method for robotic multimodal object category recognition. We formulated a new active perception method on the basis of the MHDP (Nakamura et al., 2011b).

First, we proposed an action selection method based on the IG criterion and showed that IG is an optimal criterion for active perception from the viewpoint of reducing the expected KL divergence between the final and current recognition states. Second, we proved that the IG has a submodular property and reduced the sequential active perception problem to a submodular maximization problem. Third, we derived a Monte Carlo approximation method for evaluating IG efficiently and made the action selection method executable. Given the theoretical results, we proposed to use the greedy and lazy greedy algorithms for selecting a set of actions for active perception. It is important to note that all of the three theoretical contributions mentioned above were naturally derived from the characteristics of the MHDP. These contributions are clearly a result of the theoretical soundness of the MHDP. In this sense, our theorems reveal a new advantage of the MHDP that other several heuristic multimodal object categorization methods do not have.

To evaluate the proposed methods empirically, we conducted experiments using an upper-torso humanoid robot and a synthetic dataset. Our results showed that the method enables the robot to actively select actions and recognize target objects quickly and accurately.

One of the most interesting points of this paper is that not only object categories but also an action selection for object recognition can be formed in an unsupervised manner. From the viewpoint of cognitive developmental robotics, providing an unsupervised learning model for bridging the development between perceptual and action systems is meaningful for shedding a new light on the computational understanding of cognitive development (Asada et al., 2009; Cangelosi and Schlesinger, 2015). It is believed that the coupling of action and perception is important for an embodied cognitive system (Pfeifer and Scheier, 2001).

The advantage of this paper compared with the related works in robotics is that our action selection method for multimodal category recognition has a clear theoretical basis and is tightly connected to the computational model for multimodal object categorization, i.e., MHDP. The theoretical basis gives the method preferable characteristics, i.e., theoretical guarantee.

However, note that the theoretical guarantee is satisfied only when IG is correctly estimated. We assumed that outcome of each action is deterministic and fully observable when we apply the theory of submodular optimization to active perception in multimodal categorization. However, observations *X*^*m*^ and IG are measured somehow probabilistically because of real-world uncertainty and Monte Carlo approximation. For example, IG is approximately estimated at each step of the greedy and lazy greedy algorithms. Theoretically, given this approximation in evaluating the objective being maximized, the (1−1/*e*) bound no longer holds. Streeter et al. proposed to introduce an additional penalty based on a function approximation (Streeter and Golovin, 2009). Golovin et al. extended submodularity to adaptive submodularity to consider stochastic property (Golovin and Krause, 2011). Though we discussed the proposed method from the viewpoint of submodular optimization, this algorithm can be regarded as a version of the sequential information maximization, more specifically (Chen et al., 2015). Extending our idea by referring the adaptive submodularity and/or the sequential information maximization, and update our method is our future challenge.

We assumed that each action requires same cost, and tried to reduce the number of actions in active perception, i.e., to maximize the performance of perception with the fixed number of actions. However, practically, each action, e.g., shake, hit and look at, requires different duration and different energy. Therefore, practical cost is not always the number of actions, but total cost of actions. Zhang et al. (2017) tried to deal with this problem in the context of multimodal object identification. This problem leads us a knapsack problem-like formulation. This type of submodular optimization has been studied by many researchers (Streeter and Golovin, 2009; Zhou et al., 2013). Our method will be able to be extended in the similar way.

In addition to active perception, active “learning/exploration” for multimodal categorization is also an important research topic. It takes a longer time for a robot to gather multimodal information to form multimodal object categories from a massive number of daily objects than it does to recognize a new object. If a robot can notice that “the object is obviously a sample of learned category,” the robot need not obtain knowledge about object categories from such an object. In contrast, if a target object appears to be completely new to the robot, the robot should carefully interact with the object to obtain multimodal information from the object. Such a scenario will be achieved by developing an active “learning/exploration” method for multimodal categorization. It is likely that such a method will be able to be obtained by extending our proposed active perception method.

Considering more complex categorization scenario is our future challenge. For example, Schenck et al. (2014) is dealing with the more complex categorization scenario, i.e., 36 plastic containers with identical shape and 3 colors, 4 types of contents, and 3 different amounts of those contents. In this paper, we used MHDP which assumes an object is classified into a single object category and infers the posterior distribution over categories. When we consider human cognition, we can find that object categories have more complex characteristics. For example, object categories have a hierarchical structure, an object is categorized into several classes, and they have different modality-dependency based on the types of categories. Unsupervised machine learning methods for such complex categorization problem have proposed by several researchers based on hierarchical Bayesian models (Griffiths and Ghahramani, 2006; Ando et al., 2013; Nakamura et al., 2015). Theoretically, the main assumption we used was that the MHDP is a hierarchical Bayesian model and action selection is corresponding to obtaining an observation which is a probabilistic variable on the leaf node of its graphical model. Therefore, by applying the same idea to the more complex categorization methods, we will be able to extend our theory to more complex categorization problems. This is on of our future works.

Another challenge lies in feature representation for multimodal categorization. The MHDP assumed that observations are given as bag-of-features representations. However, there are many kinds of feature representations for visual, auditory and haptic information. In particular, the feature extraction capability of deep neural networks is gathering attention, recently. Theoretically, our main theorems do not depend on the type of emission distributions, i.e., bag-of-features representations. It is likely that the same approach can be used even when a multimodal categorization method uses different feature representations, e.g., the features in the last hidden layer of a pre-trained deep neural network. This extension is also a part of our future challenges.

In addition, the MHDP model treated in this paper assumed that an action for perception is related to only one modality, e.g., grasping only corresponds to *m*^*h*^. However, in reality, when we interact with an object with a specific action, e.g., grasping, shaking, or hitting, we obtain rich information related to various modalities. For example, when we shake a box to obtain auditory information, we also unwittingly obtain haptic information and information about its weight. The tight linkage between the modality information and an action is a type of approximation taken in this research. An extension of our model and the MHDP to a model that can treat actions that are related to various modalities is also a task for our future work.

## Author contributions

The main theory was developed by TaT. The experiments were conceived by RY. The data were analyzed by RY and ToT with help of TaT. The manuscript was written by TaT.

### Conflict of interest statement

The authors declare that the research was conducted in the absence of any commercial or financial relationships that could be construed as a potential conflict of interest.

## Footnotes

^1^HDP is a nonparametric Bayesian extension of latent Dirichlet allocation (LDA) (Blei et al., 2003), which has been widely used for document-word clustering. The nonparametric Bayesian extension allows HDP to estimate the number of topics, i.e., clusters, as well.

^2^We can consider an extension of this problem by introducing different cost to each action, i.e., different action requires different time and energy. However, for simplicity, this paper focuses on the problem in which cost for each action is the same.

^3^The collected datasets for this experiment can be found in GitHub: https://github.com/tanichu/data-active-perception-hmdp

^4^Note that currently we don't have a good criteria of KL divergence to determine whether performing further actions are necessary or not.

## Appendix A: proof of the optimality of the proposed active perception strategy

In this appendix, we show that the proposed active perception strategy, which maximizes the expected KL divergence between the current state and the posterior distribution of **z**_*j*_ after a selected set of actions, minimizes the expected KL divergence between the next and final states.

(A1) $$\begin{array}{l} A_{j}^{*}=\underset{A\in F_{L}^{m_{o}{\;}_{j}}}{\text{argmin}}E_{X_{j}^{M\setminus m_{o}{\;}_{j}}|X_{j}^{m_{o}{\;}_{j}}}[\text{KL }(P(z_{j}|X_{j}^{M}),P(z_{j}|X_{j}^{A\cup m_{o}{\;}_{j}}))]\\ \;=\underset{A\in F_{L}^{m_{o}{\;}_{j}}}{\text{argmin}}\sum_{X_{j}^{M\backslash m_{o}{\;}_{j}}}\sum_{z_{j}}[P(X_{j}^{M\setminus m_{o}{\;}_{j}}|X_{j}^{m_{o}{\;}_{j}})P(z_{j}|X_{j}^{M})\\ \;\log\frac{P(z_{j}|X_{j}^{M})}{P(z_{j}|X_{j}^{m_{o}{\;}_{j}},X_{j}^{A})}] \end{array}$$

The numerator inside of the log function does not depend on **A**. Therefore, the term related to the numerator can be deleted. In addition, by negating the remaining term, we obtain

(A2) $$\begin{array}{l} (\text{A}1)=\underset{A\in F_{L}^{m_{o}{\;}_{j}}}{\text{argmin}}\sum_{X_{j}^{M\backslash m_{o}{\;}_{j}}}\sum_{z_{j}}\left[P(X_{j}^{M\setminus m_{o}{\;}_{j}}|X_{j}^{m_{o}{\;}_{j}}\right)P(z_{j}|X_{j}^{M})\\ \;\log P(z_{j}|X_{j}^{m_{o}{\;}_{j}},X_{j}^{A})]\\ \;=\underset{A\in F_{L}^{m_{o}{\;}_{j}}}{\text{argmin}}\sum_{X_{j}^{M\backslash m_{o}{\;}_{j}}}\sum_{z_{j}}\left[P(z_{j},X_{j}^{M\setminus m_{o}{\;}_{j}}|X_{j}^{m_{o}{\;}_{j}}\right)\\ \;\log P(z_{j}|X_{j}^{m_{o}{\;}_{j}},X_{j}^{A})]. \end{array}$$

By marginalizing $X_{j}^{M\setminus (m_{o}{\;}_{j}\cup A)}$ from (2), we obtain

$$\begin{array}{l} \left(\text{A}2)=\underset{A\in F_{L}^{m_{o}{\;}_{j}}}{\text{argmin}}\sum_{X_{j}^{A}}\sum_{z_{j}}P(z_{j},X_{j}^{A}|X_{j}^{m_{o}{\;}_{j}})\log P(z_{j}|X_{j}^{m_{o}{\;}_{j}},X_{j}^{A}\right)\\ \;=\underset{A\in F_{L}^{m_{o}{\;}_{j}}}{\text{argmin}}[(\sum_{X_{j}^{A}}\sum_{z_{j}}P(z_{j},X_{j}^{A}|X_{j}^{m_{o}{\;}_{j}})\log P(z_{j}|X_{j}^{m_{o}},X_{j}^{A}))\\ \;\times (-\underset{constant\;w.r.t.\;A}{\underset{︸}{\sum_{z_{j}}P(z_{j}|X_{j}^{m_{o}{\;}_{j}})\log P(z_{j}|X_{j}^{m_{o}{\;}_{j}})}})]\\ \;=\underset{A\in F_{L}^{m_{o}{\;}_{j}}}{\text{argmin}}[\sum_{X_{j}^{A}}\sum_{z_{j}}\left[P(X_{j}^{A}|X_{j}^{m_{o}{\;}_{j}})P(z_{j}|X_{j}^{m_{o}{\;}_{j}},X_{j}^{A}\right)\\ \;\log P(z_{j}|X_{j}^{m_{o}{\;}_{j}},X_{j}^{A})]\;-\sum_{X_{j}^{A}}\sum_{z_{j}}\left[P(X_{j}^{A}|X_{j}^{m_{o}{\;}_{j}})P(z_{j}|X_{j}^{m_{o}{\;}_{j}},X_{j}^{A}\right)\\ \;\log P(z_{j}|X_{j}^{m_{o}{\;}_{j}})]]\\ \;=\underset{A\in F_{L}^{m_{o}{\;}_{j}}}{\text{argmin}}\sum_{X_{j}^{A}}\sum_{z_{j}}[P(X_{j}^{A}|X_{j}^{m_{o}{\;}_{j}})\text{KL }(P(z_{j}|X_{j}^{m_{o}{\;}_{j}},X_{j}^{A}),\\ \;P(z_{j}|X_{j}^{m_{o}{\;}_{j}}))]\\ \;=\underset{A\in F_{L}^{m_{o}{\;}_{j}}}{\text{argmin}}E_{X_{j}^{A}|X_{j}^{m_{o}{\;}_{j}}}[\text{KL }(P(z_{j}|X_{j}^{A\cup m_{o}{\;}_{j}}),P(z_{j}|X_{j}^{m_{o}{\;}_{j}}))]. \end{array}$$
